# Competitive interaction with keystone taxa induced negative priming under biochar amendments

**DOI:** 10.1186/s40168-019-0693-7

**Published:** 2019-05-20

**Authors:** Lijun Chen, Yuji Jiang, Chao Liang, Yu Luo, Qinsong Xu, Cheng Han, Qiguo Zhao, Bo Sun

**Affiliations:** 1 State Key Laboratory of Soil and Sustainable Agriculture, Institute of Soil Science, Chinese Academy of Sciences, No. 71 East Beijing Road, Nanjing, 210008 China; 2 Institute of Applied Ecology, Chinese Academy of Sciences, Shenyang, 110016 China; 3 Zhejiang Provincial Key Laboratory of Agricultural Resources and Environment, Institute of Soil and Water Resources and Environmental Science, Zhejiang University, Hangzhou, 310058 China; 4 College of Life Science, Nanjing Normal University, Nanjing, 210023 China; 5 School of Geography Science, Nanjing Normal University, Nanjing, 210023 China; 6 University of Chinese Academy of Sciences, Beijing, 100049 China

**Keywords:** Biochar, Bacterial and fungal diversity, Competitive interaction, DNA-SIP microcosms, Keystone taxa, Soil organic carbon mineralization

## Abstract

**Background:**

Biochar amendments have been widely proposed as a conventional and efficient strategy to promote soil organic carbon (SOC) sequestration via negative priming. Unfortunately, the extent and biological mechanisms responsible for biochar-induced negative priming are still not fully understood. Despite traditional explanations focused on the environmental filtering mechanisms of biochar amendments on microbial biomass and community composition underlying the priming effect on SOC dynamics, whether and how a biochar-induced competitive interaction with keystone taxa determines SOC mineralization in natural ecosystems has been minimally explored.

**Results:**

Here, we paid particular attention to the relationships between the diversity and network structure of soil bacterial and fungal communities and SOC mineralization. A 3-year field experiment was conducted comprising five treatments: no fertilization, conventional fertilization, and conventional fertilization with three rates of biochar amendments. Biochar amendments considerably increased soil moisture capacity and pH and subsequently shaped the composition and co-occurrence networks of soil bacterial and fungal communities. Importantly, network analysis revealed that the biochar amendments triggered the competitive interaction with putative keystone taxa in the bacterial and fungal networks. Structural equation modeling suggested that the competitive interaction with keystone taxa promoted bacterial and fungal diversity and consequently reduced carbohydrate catabolism and soil metabolic quotient. Stable isotope probing incubations further provided consistent evidence of competition by keystone taxa with the increases in bacterial and fungal diversity under the biochar amendments.

**Conclusions:**

We found that biochar-induced competition with keystone taxa stimulated the bacterial and fungal diversity and consequently decreased SOC mineralization. The comprehensive understanding of the unexplored biological mechanisms underlying the biochar-induced negative priming may provide crucial implications for enabling SOC sequestration.

**Electronic supplementary material:**

The online version of this article (10.1186/s40168-019-0693-7) contains supplementary material, which is available to authorized users.

## Background

Soil organic carbon (SOC) sequestration is of fundamental importance in agricultural soils, because it mitigates atmospheric carbon dioxide (CO_2_) emissions and enhances soil fertility [1]. In this context, biochar application has been confirmed as an efficient way to mediate SOC sequestration in agricultural soils [2]. Biochar has the potential to regulate native SOC mineralization via positive, neutral, or negative priming effects [3]. These uncertainties in SOC mineralization induced by the biochar amendments could be attributed to the remarkable shifts in microbial abundance and composition [4–6]. Bulk of traditional explanatory studies have demonstrated that soil physicochemical conditions altered by the biochar amendments, especially pH and hydrologic characteristics, play a crucial role in controlling the biomass and composition of soil microbial communities [2]. Biochar imprints the alterations in the composition of soil microbial communities that emerge from the significantly increased ratios of fungi to bacteria and Gram-positive bacteria to Gram-negative bacteria [7, 8]. Soil microbial communities fuel SOC storage directly through their catabolic decomposition and anabolic synthesis and facilitate negative priming in terrestrial ecosystems [9, 10]. Alternatively, negative priming may occur owing to the decreased turnover rate of the existing SOC by suppressing microbial activity [11]. Until now, the essential questions about the biological mechanisms of biochar-induced negative priming by microbial communities are still far from being adequately addressed.

Network-based analytical approach is a powerful tool to infer the microbial interactions and keystone taxa of the complex networks in natural environments [12]. Microbial keystone taxa are highly connected taxa that individually or in a guild show great explanatory power of network structure and functioning irrespective of their abundance [13]. The taxa interactions and keystone taxa in the networks are often pertinent to the major shifts in the whole community structure [14]. It is reasonable to suppose, therefore, that biochar exerts important influences on the microbial interactions and keystone taxa of the bacterial and fungal co-occurrence networks [15]. Biochar has been reported to substantially improve microbial diversity, suggesting the enhanced growth of a few novel bacterial groups with previously low relative abundance [16]. In particular, the new emerging keystone taxa were highly connected in the microbiome networks, which contribute largely to modulating microbial diversity and community structure and explain microbiome compositional turnover better than whole individuals combined [14]. Accumulating theoretical and empirical evidence suggests the importance of competition in stimulating taxa co-existence and diversity through evolutionary processes [17, 18]. Hitherto, few reports have yet highlighted the mechanisms of competitive interaction accompanied by keystone taxa responsible for microbial diversity. Specifically, there is a need for further experimental evidence to verify the competitive interaction with keystone taxa in the microbial networks.

To predict SOC dynamics, it is critical to understand how the bacterial and fungal communities change in terms of their richness and diversity under the biochar amendments. So far, scarce attention has been paid to the roles of microbial diversity in mediating SOC dynamics. Despite understanding that the impact of microbial richness on community functioning depends ultimately on the traits of keystone taxa in a microbiome [19], there is a little prediction of how competitive interaction mediates the diversity-functioning relationships. The direction and extent to which microbial interactions affect diversity-functioning relationships are still a matter of considerable controversy. Intense competitive interactions with keystone taxa can attenuate or reverse diversity-functioning relationships [20]. The omission of microbial interactions from biochar-induced negative priming posits a key uncertainty for projecting the magnitude of SOC sequestration.

Based on the literature cited above, we aimed to gain a mechanistic understanding of how biochar-induced competitive interaction with keystone taxa determined SOC mineralization. Specifically, the present study was structured to clarify three questions: (1) Do the biochar amendments affect the biomass, composition, and co-occurrence networks of soil bacterial and fungal communities? (2) Does the competitive interaction with keystone taxa exhibit a substantial impact on soil bacterial and fungal diversity under the biochar amendments? If so, then (3) what is the biological mechanism responsible for biochar-mediating SOC mineralization via priming effects? To achieve these goals, we conducted a 3-year field experiment under three rates of biochar amendments along with two non-biochar amendments. We observed that the competitive interaction with keystone taxa in the networks promoted bacterial and fungal diversity and then decreased soil mineralization. Our work describes new entry points for the undefined biological mechanisms of biochar-induced negative priming effect on SOC mineralization.

## Results

### Soil physicochemical properties and SOC mineralization

**Fig. 1:**
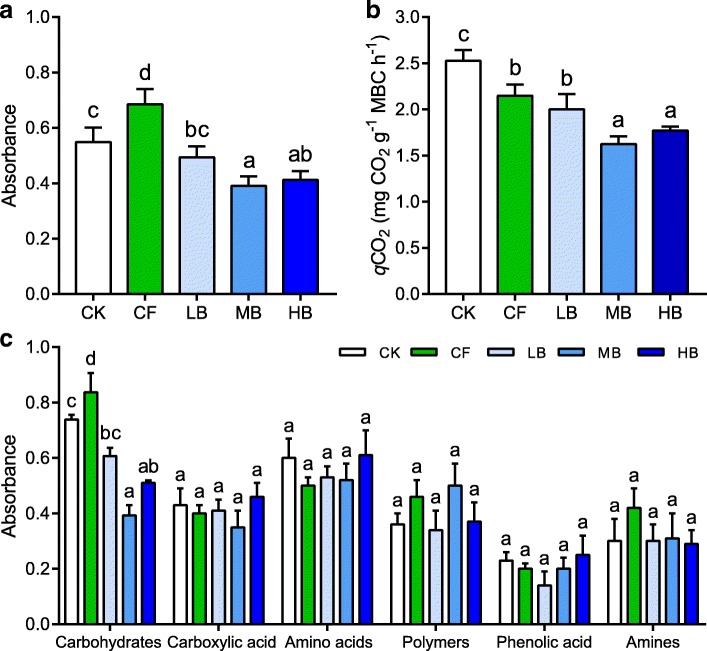
Effects of biochar amendments on microbial carbon metabolic activities (**a**) and soil metabolic quotient (**b**; *q*CO_2_) in the flied experiment. Carbon source utilization rates based on six specific carbon families are analyzed by the Biolog EcoPlates including carbohydrates, carboxylic acids, amino acids, polymers, phenolic acid, and amines/amides (**c**). Microbial carbon metabolism is reflected by the average well color development (AWCD). Bars with different lowercase letters indicate statistical significant differences (*P* < 0.05) as revealed by Bonferroni’s post hoc tests. CK, no fertilizer; CF, conventional fertilization; LB, low biochar with 2400 kg ha^−1^ year^−1^; MB, medium biochar with 7200 kg ha^−1^ year^−1^; HB, high biochar with 12,000 kg ha^−1^ year^−1^

Biochar amendments significantly affected soil physicochemical conditions (*F*_(4, 11)_ = 2.67–73.56, *P* < 0.05). Soil pH, SOC, total nitrogen, total phosphorus, available potassium, and available phosphorus were significantly elevated by the medium and high biochar (MB and HB) amendments relative to conventional fertilization (CF) (Additional file 1: Table S1, *P* < 0.05). Similarly, total potassium ranged from 20.74 to 23.55 g kg^−1^, with a significant increase under the HB amendment (*P* < 0.05). However, bulk density and available nitrogen under the MB and HB amendments were significantly lower than those under the CF treatment (Additional file 1: Table S1, *P* < 0.05). In comparison, there were no significant effects of biochar obtained for cation exchange capacity (*P* = 0.982). Soil moisture capacity (SMC) under the three biochar amendments increased sharply when soil water suction was higher than 0.03 MPa (Additional file 1: Figure S1, *P* < 0.05). The diameter–θ curves showed that the biochar amendments increased the size of soil effective pores to larger than 0.1 μm (Additional file 1: Figure S1). The results of soil water characteristic curve indicated that the water retention capacity gradually increased with the increasing rates of biochar amendments. SOC mineralization was estimated by both soil metabolic quotient (*q*CO_2_) and microbial carbon metabolism represented by average well color development (AWCD). On average, the values of AWCD and *q*CO_2_ significantly decreased by 36.8% and 16.3% under the biochar amendments compared to the CF treatment, respectively (Fig. 1, *P* < 0.05), and the MB amendment was characterized by the lowest value of AWCD and *q*CO_2_. Carbohydrate utilization followed a similar trend to the AWCD value of whole plate (Fig. 1, *P* < 0.05). However, no significant differences were observed in the utilization of other five carbon sources, including carboxylic acid, amino acids, phenolic acid, amines, or polymers.

### The biomass, diversity, and composition of soil bacterial and fungal communities

Soil samples were subjected to phospholipid fatty acid (PLFA) analysis for determining microbial biomass. Microbial biomass measured as total PLFA was significantly higher under the MB and HB amendments than under the CF treatment (Additional file 1: Figure S2, *P* < 0.05). Similar to total PLFA, the biomass of microbial-specific groups significantly increased under the MB and HB treatments in terms of bacteria (21.3% and 34.2%), Gram-negative bacteria (29.1% and 47.5%), Gram-positive bacteria (9.1% and 15.1%), actinomycetes (13.8% and 17.8%), and fungi (14.2% and 31.8%) (Additional file 1: Figure S2, *P* < 0.05).

**Fig. 2:**
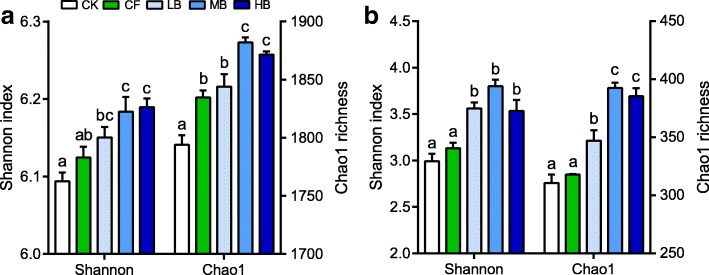
Biochar amendments alter the bacterial (**a**) and fungal (**b**) diversity in the field experiment. Calculation of the Shannon index and Chao1 richness is based on OTU tables rarified to the same sequencing depth. Bars with different lowercase letters are significantly different (*P* < 0.05) by Bonferroni’s post hoc tests. CK, no fertilizer; CF, conventional fertilization; LB, low biochar with 2400 kg ha^−1^ year^−1^; MB, medium biochar with 7200 kg ha^−1^ year^−1^; HB, high biochar with 12,000 kg ha^−1^ year^−1^

The diversity and compositions of soil bacterial and fungal communities were detected by Illumina sequencing of 16S *rRNA* and ITS gene amplicons. After rarefaction to equal sequencing depth, we obtained a total of 409,125 and 446,565 high-quality bacterial and fungal sequences with an average of 1652 bacterial and 298 fungal OTUs across all samples. The MB and HB amendments significantly increased bacterial and fungal diversity estimated by the Shannon index and Chao1 richness compared with the no fertilizer (CK) and CF treatments (Fig. 2). The bacterial communities consisted mainly of *Actinobacteria* (31.2%), *Alphaproteobacteria* (20.4%), *Chloroflexi* (8.73%), *Acidobacteria* (7.2%), *Betaproteobacteria* (6.3%), *Bacteroidetes* (5.4%), *Gammaproteobacteria* (5.0%), *Gemmatimonadetes* (4.6%), and *Firmicutes* (3.5%) (Additional file 1: Figure S3). The principal coordinate analysis of Bray-Curtis distances displayed that the bacterial community compositions under the biochar amendments were significantly (*P* < 0.01) clustered together on the basis of their taxonomy (Additional file 1: Figure S4). When considering the relative bacterial abundances, *Actinobacteria*, *Gammaproteobacteria*, and *Gemmatimonadetes* were enriched under the biochar amendments, while *Alphaproteobacteria* and *Acidobacteria* were overrepresented under the CK and CF treatments (*P* < 0.05). The fungal community compositions were dominated by *Ascomycota* (89.3%) belonging to the classes *Sordariomycetes* (60.8%), *Eurotiomycetes* (23.0%), and *Dothideomycetes* (3.2%), followed by the rare phyla *Basidiomycota* (8.1%) and *Zygomycota* (2.0%) (Additional file 1: Figure S3). Similar to the bacterial community, the comparison of the fungal community compositions by principal coordinates analysis revealed a significant (*P* < 0.01) separation among the biochar amendments and two compartments (CK and CF) (Additional file 1: Figure S4). At the phylum/class level, the taxonomical differences resulted primarily from the higher abundance of *Basidiomycota* but the lower abundance of *Sordariomycetes* (*P* < 0.05). Overall, the similarities of soil bacterial and fungal community composition within the biochar amendments were significantly (*P* < 0.05) higher than those between the biochar amendments and their counterparts including CK and CF (Additional file 1: Figure S4).

### Bacterial and fungal co-occurrence networks

**Fig. 3:**
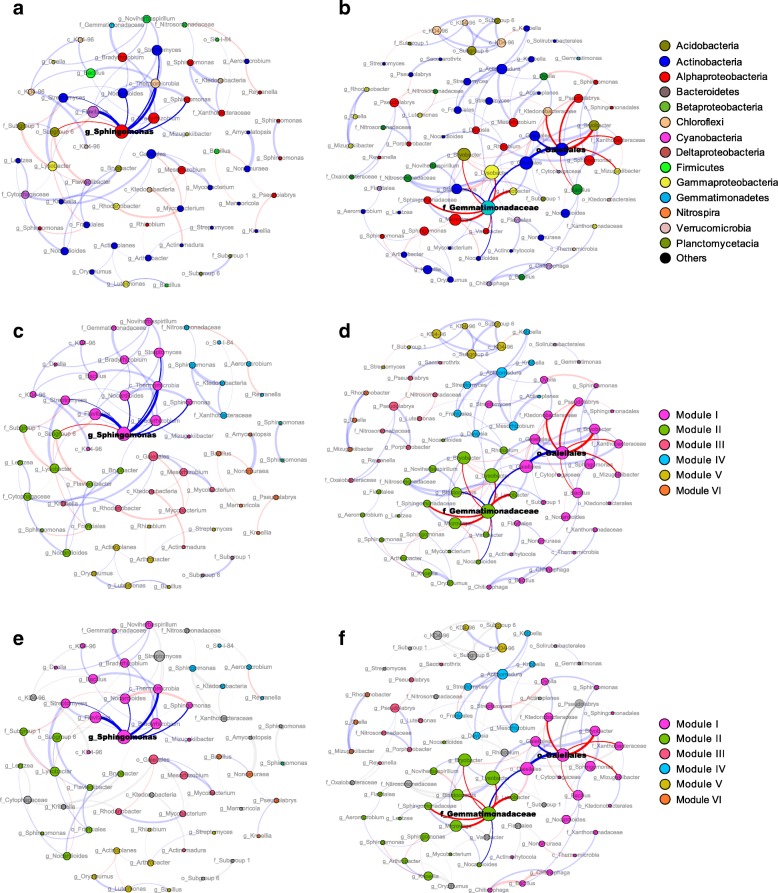
The bacterial co-occurrence networks under the non-amended (CK and CF) and biochar-amended (LB, MB, and HB) treatments based on correlation analysis in the field experiment and stable isotope probing (SIP) microcosms. A connection stands for a strong (Spearman’s *P* > 0.8) and significant (*P* value < 0.01) correlation for the non-amended and biochar-amended treatments. The non-amended (**a**, **c**) and biochar-amended (**b**, **d**) networks in the field experiment are colored by the bacterial phyla/classes and modules, respectively. The bacterial modules I–VI in the non-amended and biochar-amended networks were the six clusters of closely interconnected nodes. The bacterial keystone taxa (module hubs) and their connected edges in the networks are in bold. The size of each node is proportional to the number of connections (degree), and the thickness of each connection between two nodes (edge) is proportional to the value of Spearman’s correlation coefficients. The blue edges indicate positive interactions between two bacterial nodes, while red edges indicate negative interactions. The non-amended (**e**) and biochar-amended networks (**f**) in the SIP microcosms are colored by bacterial phyla/classes. The gray nodes and edges are not detected

To identify the co-occurrence patterns of soil bacterial and fungal communities and niche partition in the non-amended (CK and CF) and biochar-amended (LB, MB, and HB) treatments, we next constructed the bacterial and fungal networks in which nodes and links were calculated by the robustness of the co-occurrence scores. Although there were more positive correlations in the bacterial and fungal networks regardless of treatments, the ratios of negative correlations to positive correlations were increased under the biochar amendments (Fig. 3, Additional file 1: Table S2). Multiple topological properties of the bacterial and fungal co-occurrence patterns pronouncedly varied in the biochar-amended networks in terms of the numbers of nodes and edges, average connectivity, and average clustering coefficient (Additional file 1: Table S2).

**Fig. 4:**
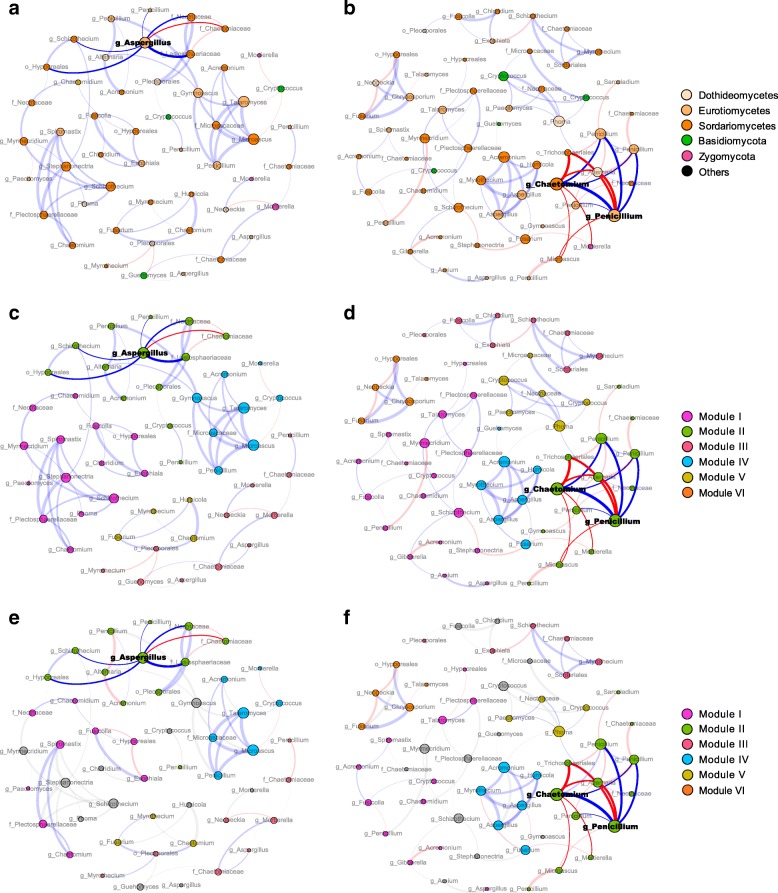
The fungal co-occurrence networks under the non-amended (CK and CF) and biochar-amended (LB, MB, and HB) treatments based on correlation analysis in the field experiment and stable isotope probing (SIP) microcosms. A connection stands for a strong (Spearman’s *P* > 0.8) and significant (*P* value < 0.01) correlation for the non-amended and biochar-amended treatments. The non-amended (**a**, **c**) and biochar-amended (**b**, **d**) networks in the field experiment are colored by the fungal phyla/classes and modules, respectively. The fungal modules I–VI in the non-amended and biochar-amended networks were the six clusters of closely interconnected nodes. The fungal keystone taxa (module hubs) and their connected edges in the networks are in bold. The size of each node is proportional to the number of connections (degree), and the thickness of each connection between two nodes (edge) is proportional to the value of Spearman’s correlation coefficients. The blue edges indicate positive interactions between two fungal nodes, while red edges indicate negative interactions. The non-amended (**e**) and biochar-amended networks (**f**) in the SIP microcosms are colored by fungal phyla/classes. The gray nodes and edges are not detected

**Fig. 5:**
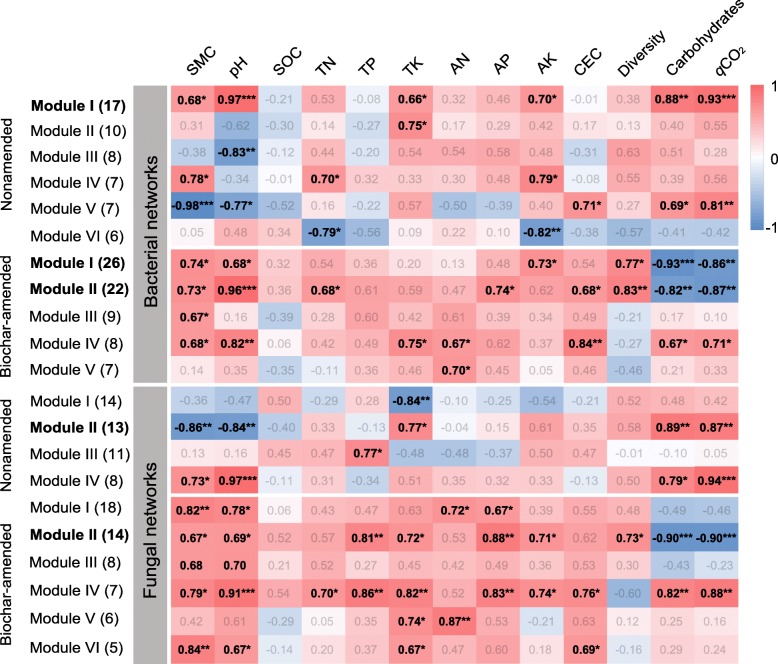
Correlation coefficients between module eigengenes, soil properties and carbohydrate catabolism, and soil metabolic quotient (*q*CO_2_) under non-amended and biochar-amended treatments. The numbers in parentheses indicate the nodes observed in each module. The bacterial and fungal diversities are represented by the Shannon index based on the rarified same sequencing depth. The bacterial and fungal networks are represented by the module eigengenes that are significantly related to diversity and carbohydrate metabolism. The bacterial and fungal modules in the non-amended and biochar-amended networks are the clusters of closely interconnected nodes. The bacterial and fungal modules with keystone taxa in the non-amended and biochar-amended networks are in bold. Bold values denote the significant relationships. SMC, soil moisture capacity; SOC, soil organic carbon; TN, total nitrogen; TP, total phosphorus; TK, total potassium; AN, available nitrogen; AP, available phosphorus; AK, available potassium; CEC, cation exchange capacity. ***P < 0.001; **P < 0.01; *P < 0.05

The bacterial and fungal networks were clustered into modules that could be examined to find significant module-trait relationships. Our results indicated that both bacterial and fungal networks could be decomposed into smaller coherent modules and that their eigengenes were strongly correlated with SMC and pH (Figs. 3, 4 and 5). The bacterial modules I and V and fungal modules II and IV were positively correlated with carbohydrate utilization and *q*CO_2_ in the non-amended networks (Fig. 5). However, the bacterial modules I and II and fungal module II showed negative correlations with carbohydrate catabolism and *q*CO_2_ in the biochar-amended networks. Noteworthy, the bacterial modules I and II and fungal module II were positively associated with bacterial and fungal diversity, respectively (Fig. 5). Furthermore, the individual nodes displayed discrepant roles in the microbial networks according to the within-module connectivity *Z* and among-module connectivity *P*. For the non-amended network, the bacterial genus *Sphingomonas* (*Alphaproteobacteria*) and the fungal genus *Aspergillus* (*Eurotiomycetes*) were detected as the module hubs. Intriguingly, the statistically identified keystone taxa displayed positive relationships with connected members in their own module, as well as with carbohydrate catabolism and *q*CO_2_ (Figs. 3 and 4, Table 1). For the biochar-amended network, the bacterial genera *Arthrobacter* (*Actinobacteria*) and *Gemmatimonadaceae* (*Gemmatimonadetes*) and fungal genera *Chaetomium* (*Sordariomycetes*) and *Penicillium* (*Eurotiomycetes*) were analogously designated as module hubs. However, these keystone taxa were of intensely negative relevance for the linked nodes in the individual modules and for carbohydrate catabolism and *q*CO_2_ (Figs. 3 and 4, Table 1). Collectively, the results suggested that these particular nodes were mandatory to summarize a module related to carbohydrate catabolism and *q*CO_2_.

**Table 1:** The keystone taxa in the bacterial and fungal networks under non-amended and biochar-amended treatments

Network	OTU ID	Module	Degree	Cluster coefficient	Abundance (%)^a^		Phylum/class	Affiliation	*Z* ^b^	*P* ^b^	Diversity	Carbohydrate	*q*CO_2_
					Field	SIP incubation							
Bacteria													
Non-amended	OTU2131	Module I	8	0.22	0.25	1.15	*Alphaproteobacteria*	*Sphingomonas*	2.67	0.22	**−** 0.357	0.627*	0.602*
Biochar-amended	OTU717	Module I	8	0.23	0.20	12.39	*Actinobacteria*	*Arthrobacter*	3.12	0	0.798***	**−** 0.879***	**−** 0.756**
	OTU316	Module II	9	0.29	0.09	0.18	*Gemmatimonadetes*	*Gemmatimonadaceae*	2.95	0.19	0.617*	**−** 0.841***	**−** 0.789***
Fungi													
Non-amended	OTU148	Module II	6	0.35	0.05	5.44	*Eurotiomycetes*	*Aspergillus*	2.53	0	**−** 0.479	0.763**	0.762**
Biochar-amended	OTU430	Module II	7	0.57	0.26	0.09	*Sordariomycetes*	*Chaetomium*	2.57	0	0.832***	− 0.722**	**−** 0.654**
	OTU516	Module II	7	0.62	0.14	7.14	*Eurotiomycetes*	*Penicillium*	2.57	0	0.609*	− 0.722**	**−** 0.642**

*q*CO_2_, soil metabolic quotient. *P < 0.05; **P < 0.01; ***P < 0.001.

^a^Abundance of keystone species in the field experiment and stable isotope probing (SIP) microcosms

^b^The topological role of each node is determined based on two properties: the within-module connectivity *Z* and the among-module connectivity *P*

### Soil properties and microbial community affected SOC dynamics

**Fig. 6:**
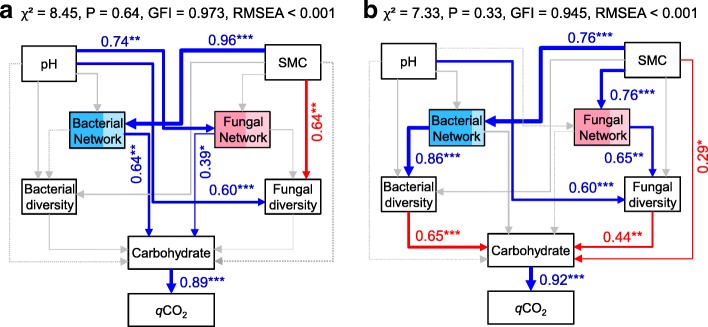
The impacts of soil properties and bacterial and fungal community on carbohydrate metabolism and soil metabolic quotient (*q*CO_2_) as estimated using the structural equation modeling under the non-amended (**a**) and biochar-amended (**b**) treatments. Blue lines indicate positive relationships, while red lines indicate negative relationships. The bacterial and fungal diversities are represented by the Shannon indexes based on the rarified same sequencing depth, and the bacterial and fungal networks are represented by the module eigengenes that are significantly related to diversity and carbohydrate metabolism. The contributions of the module with or without keystone taxa in the bacterial (blue rectangle) and fungal networks (red rectangle) were colored by dark or light, respectively. The width of arrows indicates the strength of significant standardized path coefficients (*P* < 0.05). Paths with non-significant coefficients are presented as gray lines. SMC, soil moisture capacity. ***P < 0.001; **P < 0.01; *P < 0.05

The diversity and richness of soil bacterial and fungal communities were positively correlated with SMC and pH (*r* = 0.732~0.937, *P* < 0.01), but were negatively related to carbohydrate utilization and *q*CO_2_ (*r* = − 0.927 to − 0.720, *P* < 0.01) (Additional file 1: Table S3). However, the bacterial and fungal biomasses showed no significant correlations with carbohydrate catabolism and *q*CO_2_ (*r* = − 0.490 to − 0.271, *P* > 0.05). Random forest modeling was used to evaluate the potential important predictors of carbohydrate catabolism and *q*CO_2_. Overall, we found that SMC and pH were the two main determinants of soil properties for carbohydrate utilization and *q*CO_2_ (Additional file 1: Figure S5). The mean square error increased 13.6% and 15.7% for carbohydrate utilization and *q*CO_2_, respectively, when removing the predictor of SMC, and increased 10.9% and 13.3%, respectively, when removing the predictor of soil pH. The bacterial and fungal diversity (Shannon index) contributed more pronounced effects to carbohydrate utilization and *q*CO_2_ than the networks (module eigengenes) (Additional file 1: Figure S5). Structural equation modeling (SEM) was further used to evaluate the direct and indirect impacts of soil properties and soil bacterial and fungal communities on SOC mineralization under the non-amended and biochar-amended treatments. Soil bacterial and fungal networks (module eigengenes) were positively related to carbohydrate catabolism under the non-amended treatments (Fig. 6). We observed that SMC showed a directly negative influence on carbohydrate catabolism under the biochar-amended treatments. Importantly, the modules with keystone taxa may contribute more to soil bacterial and fungal networks than those without keystone taxa (Fig. 6). The bacterial and fungal diversities (Shannon index) were positively correlated with the networks (module eigengenes), but were negatively associated with carbohydrate catabolism under the biochar-amended treatments. SEM suggested that bacterial diversity exhibited a larger impact on carbohydrate catabolism than fungal diversity (Fig. 6).

### SIP incubations

We conducted SIP experiments to trace the bacterial and fungal taxa capable of utilizing carbohydrates in the non-amended and biochar-amended networks. The targeted bacterial and fungal populations were successfully labeled during incubations with ^13^C-glucose. The bacterial and fungal communities were less diverse in the labeled libraries of ^13^C-incubated samples than in those of ^12^C-controls (Additional file 1: Figure S6), suggesting that only a portion of the bacterial and fungal groups using glucose compared to the overall microbial community. In fact, approximately half of all detected bacterial (46.5%) and fungal (54.7%) genera were involved in glucose utilization. Furthermore, the successful targeting of carbohydrate catabolic populations presented significantly higher diversity under the biochar-amended treatments than under the non-amended treatments (Additional file 1: Figure S6, *P* < 0.05). For the bacterial community, the members of *Actinobacteria* (52.4% and 19.6%), *Alphaproteobacteria* (16.2% and 32.5%), *Gammaproteobacteria* (6.6% and 9.2%), and *Betaproteobacteria* (4.4% and 6.6%) were dominant in the labeled libraries of ^13^C-incubated samples and ^12^C-controls, respectively (Additional file 1: Figure S7). The fungal community consisted of *Ascomycota*, predominated by *Sordariomycetes* (70.0% and 52.3%), *Eurotiomycetes* (23.1% and 22.5%), and *Dothideomycetes* (1.6% and 6.1%) in the labeled libraries of ^13^C-incubated samples and ^12^C-controls, respectively (Additional file 1: Figure S7). On average, the abundance of bacterial phylum *Actinobacteria* and fungal phylum *Sordariomycetes* in the ^13^C-glucose-utilizing populations increased 1.6 and 1.2 times in the biochar-amended treatments compared to the non-amended treatments, respectively. A vast majority of the total nodes were identified as potential glucose-utilizing bacteria (80% and 84%) and fungi (76% and 79%) in the biochar non-amended and amended networks, respectively (Figs. 3 and 4, Additional file 1: Table S2). All bacterial and fungal keystone taxa in the non-amended and biochar-amended networks were detected among these potential ^13^C-glucose-utilizing populations. The bacterial and fungal module hubs in the non-amended networks exhibited positive correlations with the connected nodes in their individual modules, while those in the biochar-amended networks were negatively associated with their linked nodes (Figs. 3 and 4).

## Discussion

### Biochar amendments altered the bacterial and fungal communities

Biochar amendments as a widely proposed strategy to adjust SOC storage and improve soil fertility are a topic of growing interest and strong concern. In the short-term field experiment, we chose three rates of biochar amendments to assess the biochar effects on soil characteristics. Our results clearly revealed that biochar amendments significantly reduced bulk density, but increased soil pH, water holding capacity, and easily available substrates. Biochar amendments are expected to immediately increase the pH of adjacent soil due to the alkaline pH of biochar, which relies heavily on the nature of feedstock materials and pyrolysis temperature. Biochar made from crop residues and formed at relatively high temperature tends to have high ash contents and carbonates [21]. Biochar is found to simultaneously promote SMC and available water to microorganisms, which can be attributed mainly to the reduced bulk density and increased proportion of large voids and porosity [22]. In addition, biochar characteristics of surface charge can facilitate the transfer of water and nutrients from bulk soil into pore structure. Due to the high internal surface area and porosity, biochar enhances water holding capacity and effectively retains moist pore spaces for continued hydration of microbial populations as soils dry [23].

The increased pH and SMC under the MB and HB amendments may enhance microbial spore germination, colonization, and reproduction rates and consequently increase bacterial and fungal biomass. Given that biochar induced the changes in microbial biomass, it was extremely unlikely that such changes in abundance were spread equally across different functional groups. Changes in physicochemical properties and substrate utilization under the biochar amendments would differentially tailor the composition and network of soil bacterial and fungal communities. The PCoA combined with network analysis confirmed that the compositions and network structures of the bacterial and fungal communities in the biochar-amended soils were significantly different from those in the non-amended soils (Figs. 3 and 4, Additional file 1: Figure S4 and Table S2). Biochar amendments directly triggered the changes in the bacterial and fungal community structures by promoting the abundance of Gram-positive bacteria and saprotrophic fungi. These results suggested that differential responses of fungal and bacterial taxa to the preferred energy sources for their metabolic needs caused some microbial groups to become competitively dominant. Select Gram-positive bacteria preferentially thrive on the surface of fresh biochar and often excrete highly complex sets of lignin-degrading oxidative enzymes necessary to depolymerize the highly complex biochar-derived C [11, 24]. Additionally, saprotrophic fungi have exceptional enzymatic capabilities to degrade biochar via the production of peroxidase, phenoloxidase, and laccase [25]. In contrast to Gram-positive bacteria, saprotrophic fungi occupy a hyphal, invasive growth habit, which gives them access to effectively colonize the interior of biochar particles.

The major changes in topological and modular features of the bacterial and fungal networks were mirrored by the alterations in community composition. The coupling linkages of the bacterial and fungal networks increased in the negative direction under the biochar amendments, suggesting antagonistic or competitive interactions for substrate acquisition. The biochar-amended networks with a higher average connectivity displayed the more complex coupling among microbes. The module in the network suggested that the microbial populations within it had similar ecological niches and functional interdependences [26]. Soil pH and SMC have been interpreted as the primary predictors of topological network properties involving network complexity and modularity [15, 27]. The increased pH and SMC under the biochar treatments directly reduced a physiological constraint on soil bacteria and fungi and changed interactive relationships [28, 29]. Alternatively, soil pH is often significantly related to a variety of soil properties (e.g., nutrient availability and organic carbon characteristics) and indirectly regulates the microbial network structure [30]. Biochar amendments reshaped the distinct keystone taxa within the bacterial and fungal networks. The bacterial and fungal communities with keystone taxa indicated the complex interactions in the co-occurrence networks. The important keystone taxa in the phyla *Actinobacteria* and *Gemmatimonadetes* are capable of growing preferentially on carbon-rich refractory materials to decompose the cellulose, lignocellulose, and chitin in biochar-amended soils [31]. However, it should be noted that soil pH varied in a narrow range under the non-amended (5.71–6.02) and biochar-amended (6.14–6.57) treatments, respectively (Additional file 1: Table S1). SEM suggested that pH had a significant effect on fungal diversity rather than on bacterial diversity, when soil pH span a narrow range at nearly neutral value (Fig. 6). Bacteria and fungi exhibit fine differences in body size, metabolic activity, and dispersal potential [32], which display different responses to the narrow pH changes. The “size-plasticity” hypothesis argues that smaller individuals are less environment filtered than larger individuals, because smaller individuals are more likely to have plasticity in metabolic abilities [33, 34]. Therefore, bacteria may exist widely in such a narrow pH range, suggesting that the selection pressure of pH was invisible on the bacterial community.

### Competition with keystone taxa stimulated the bacterial and fungal diversity

To advance the explanatory and predictive understanding of SOC sequestration, it is critical to investigate how the underlying bacterial and fungal communities shift in their diversity. Traditional dichotomy supports the divergent natural selection generated by resource competition as a vital driver of rapid diversification, especially when microbial taxa share similar resource requirements or niches in the soil microenvironment [35]. Functional and phylogenetic diversity is commonly expected to be promoted by intra- and inter-specific competitive interaction in the ecological communities [36]. We found that the effects of biochar on the bacterial and fungal diversity are potentially mediated by keystone taxa in the networks (Fig. 6, Table 1). We postulated that high microbial diversity was sustained via the recruitment of particular keystone taxa from the indigenous taxa pool in the biochar-amended soils. It is generally assumed that the uneven distribution of interaction strengths is prevalent in the networks [37]. For instance, special taxa are likely to have close connections and thereby make stronger contributions to network structure and diversity relative to poorly connected taxa [38]. The highly connected keystone taxa in the networks can individually explain microbiome compositional turnover better than all taxa combined, highlighting crucial roles for maintaining the organization integrity and the functioning of the entire microbial community [39, 40]. With network topological data, the members of the genera *Arthrobacter* and *Chaetomium* categorized as keystone taxa were exclusively observed in the bacterial and fungal networks under the biochar amendments, respectively. When the biochar-amended networks were compartmentalized into modules, the bacterial and fungal keystone taxa probably displayed intense competition with contacted members in the respective modules (Figs. 3 and 4). These presumed keystone taxa, *Arthrobacter* and *Chaetomium*, have competitive traits and advantages for breaking down recalcitrant biochar-derived C and capturing limiting resources more efficiently [41, 42]. Furthermore, the genus *Penicillium* could produce antifungal toxins to kill, inhibit growth, and displace competing fungi, forming antagonistic structures in the modules. Alternatively, multiple taxa with different competitive strategies and trait expression alter their morphology, trait expression, and metabolism to persist against direct displacement or overgrowth in diverse communities [18]. Network-based scores complemented with DNA-SIP microcosms provided consistent evidence of inter-specific competition by keystone taxa with continual increases in the bacterial and fungal diversity. Collectively, these results suggested that functional keystone taxa with mutually exclusive ability may increase bacterial and fungal diversity under the biochar amendments.

### Higher bacterial and fungal diversity decreased SOC mineralization

The increases in bacterial and fungal diversity could be of ecological significance, as greatly reduced microbial carbohydrate catabolism and *q*CO_2_ were observed under the MB and HB amendments. The present study suggested that negative SOC priming was positively correlated with increased bacterial and fungal diversity. Despite soil pH and moisture have been reported as crucial drivers of SOC dynamics, soil bacterial and fungal communities are a core backbone of terrestrial SOC cycling, mediating the vast majority of SOC mineralization. There is an ongoing controversy over whether the modern ecological concept and theory of biodiversity-ecosystem functioning relationships hold true for microorganisms [43]. Essentially, the reported impacts of microbial diversity on SOC dynamics vary considerably, ranging from being negative over neutral to positive. The functional traits of keystone taxa have frequently been proposed to be of particular ecological importance to determine the relationships between taxa richness and SOC dynamics [44, 45]. The keystone taxa often serve as gatekeepers to community functions, with profound contributions to SOC transformation and sequestration [46].

Biochar can facilitate negative SOC priming in the long term due to lowering the microbial mineralization rate of natural SOC [47, 48]. The overall diversity of bacterial and fungal communities was negatively associated with microbial carbohydrate catabolism and *q*CO_2_ (Fig. 6, Additional file 1: Table S3). Theoretically, the competitive interaction in the networks can powerfully determine the direction and magnitude of the diversity-function relationships [49]. Although the role of competition was relatively weak in the entire network, the competitive interaction with keystone taxa may outweigh any positive effects of highly co-existing taxa on SOC dynamics under the biochar amendments (Figs. 3, 4 and 5). We confirmed that the biochar-induced competitive interaction with keystone taxa benefited SOC accumulation by inhibiting of carbohydrate catabolism and *q*CO_2_. In marked contrast, the non-amended networks exhibited a pairwise positive relationship with keystone taxa. Co-existing taxa with cooperative interactions may stimulate community performance relevant to increasing SOC mineralization. A new set of experimental studies with synthesized microcosms has demonstrated that high richness (more than ten species) enhances the inhibitory force of competitors with the capability to dampen SOC use efficiency [18, 50]. The soil has typically extremely high diversity in the bacterial and fungal communities, where competitive stress associated with inter-specific interactions may likewise contribute to the eventual suppression of carbon catabolic rates in the natural field systems [43, 51]. Nevertheless, it should be noteworthy that competitive relationships do not inevitably correspond to “poor” ecosystem functioning. Reduced carbon metabolism and SOC mineralization rates with increasing bacterial and fungal diversity could stimulate SOC retention under the biochar amendments. Taken together, our results favored the previous inference that the intensely competitive or antagonistic interactions could impair the community performance of SOC mineralization at high levels of diversity. More broadly, the predictions of how SOC mineralization varies with microbial diversity will enrich our knowledge of the mechanism underlying the diversity-function patterns in diverse microbial communities.

## Conclusions

In conclusion, biochar amendments induced negative priming effects on SOC sequestration by competitive interaction coupled with keystone taxa in the microbiome networks. Biochar amendments structured the competition with keystone taxa in the bacterial and fungal networks, which promoted microbial diversity and subsequently reduced carbohydrate catabolism and *q*CO_2_. Our study highlighted the important role of biochar-induced competition with keystone taxa in stimulating SOC sequestration via negative priming. This finding will stimulate the novel theoretical developments for the priming effect on SOC dynamics.

## Methods

### Field experiment description

The field experiment was conducted at the tobacco field station (35° 51′ 36″ N, 118° 37′ 48″ E), located in Linyi, Shandong Province, China. This site has a temperate monsoon climate with an annual average temperature and precipitation of 14.1 °C and 849 mm, respectively. The tested soil is characterized as a Hapli-Ustic Cambisol in the Food and Agricultural Organization (FAO) classification system. Fifteen trial plots (5 m long × 2 m wide) were laid out in a completely randomized block including five treatments with three replicates: (1) no fertilizer (CK), (2) conventional fertilization (CF), (3) low biochar with 2400 kg ha^−1^ year^−1^ (LB), (4) medium biochar with 7200 kg ha^−1^ year^−1^ (MB), and (5) high biochar with 12,000 kg ha^−1^ year^−1^ (HB). The CF treatment contained 450 kg ha^−1^ compound fertilizer (10–10–30, N–P_2_O_5_–K_2_O), 600 kg ha^−1^ fermented soybean meal, and 150 kg ha^−1^ K_2_SO_4_. Three biochar amendments were given the equal application rates of nitrogen, phosphorus, and potassium as those under the CF treatment. The biochar used in the experiment was produced from maize straw pyrolyzed at 450 °C for 48 h. The biochar had a pH value of 9.49, SOC of 915.4 g kg^−1^, total nitrogen of 2.32 g kg^−1^, total phosphorus of 3.03 g kg^−1^, total potassium of 32.94 g kg^−1^, available phosphorus of 336.24 mg kg^−1^, available potassium of 18,125 mg kg^−1^, and cation exchange capacity of 15.06 cmol kg^−1^. Tobacco (*Nicotiana tabacum* L., cultivar NC102) grown in a monoculture was transplanted in May and harvested in August since 2014. Briefly, tillage and ridging were conducted 1 week before the tobacco seedling transplanting. Healthy seedling transplanting was performed with a population density of 18,000 plants per hectare in each field plot. Technical guidelines for the management of tobacco crops were applied during the whole growth season. We manually removed the grass at the rosetting stage, snapped the tobacco flowers at the topping stage, and harvested mature leaves from bottom to top of the stalks at the maturity stage. Tobacco crops were harvested three times at weekly interval.

### Soil sampling and physicochemical properties

Soil samples from each plot were collected at a depth of 0–20 cm after the harvest in early August 2016. After field collection, fresh samples were placed on ice and immediately transported to the laboratory, where they were sieved (2 mm) to remove visible residues and then homogenized. Soil samples were subdivided into three subsamples for analyzing soil physiochemical properties, microbial communities and functions, and DNA-SIP microcosms.

Soil pH was detected by a glass electrode with water: soil ratio of 2.5:1 (*v*/*w*). SOC was determined by wet digestion using the potassium dichromate method [52]. Total nitrogen and available nitrogen were measured using the micro-Kjeldahl method and the alkaline hydrolysis diffusion method, respectively [53, 54]. Total phosphorus was digested with HF-HClO_4_ and available potassium was extracted with sodium bicarbonate, respectively, which were determined using the molybdenum-blue method [55, 56]. Total potassium was digested with HF–HClO_4_ and available potassium was extracted with ammonium acetate, respectively, which were detected by atomic absorption spectrophotometer [57]. Cation exchange capacity was measured by ammonium acetate solution at pH 7 [58]. Bulk density was determined by the standard cylinders of 43 cm^3^. Soil water characteristic curve was measured at 0, 0.0025, 0.006, 0.01, 0.03, 0.1, and 1.5 MPa using the pressure membrane meter method [59]. Soil moisture capacity (SMC) are calculated by progressive summation of pore volumes fractions based on soil water characteristic curve, accomplished by pairing each calculated pore volume fractions with the equivalent pore radius [60].

### PLFA profiles

To address question 1, we characterized microbial biomass by PLFA analysis following a modified method [61]. Briefly, total lipids were extracted from 2 g freeze-dried soil samples with a chloroform-methanol-citrate buffer mixture (1:2:0.8, *v*/*v*/*v*) and separated into neutral, glyco- and phospholipids by a silica acid column. Phospholipids were subjected to a mild alkaline methanolysis, and the fatty acid methyl esters were quantified by a HP 6890 Series gas chromatograph instrument (Hewlett Packard, Wilmington, DE, USA). Identification was performed using bacterial fatty acid standards and MIDI peak identification software (Microbial ID Inc., Newark, DE, USA). Microbial biomass was calculated by summing the abundance of specific biomarkers and expressed as nmol PLFA g^−1^ dry soil [62]. The following PLFAs were representative markers of the specific groups: Gram-negative bacteria (cyclopropyl bacteria and unsaturated PLFAs) [63], Gram-positive bacteria (iso- and anteiso-branched PLFAs) [63], actinomycetes (10Me PLFAs) [64], and fungi (18:1 ω9c and 18:2 ω6, 9c) [62]. The sum of Gram-positive bacteria, Gram-negative bacteria, and non-specific bacteria was expressed as the bacterial biomass [62].

### Illumina sequencing and bioinformatic analysis

To address question 1, we determined the diversity, composition, and network by Illumina sequencing of 16S *rRNA* and ITS genes. The samples for analyzing the microbial communities were stored at − 80 °C until DNA extraction. The DNA was extracted from 0.25 g soil samples using a PowerSoil DNA extraction kit (MoBio Laboratories Inc., Carlsbad, CA, USA) following the manufacturer’s instructions. The extracted DNA was suspended in nuclease-free TE buffer, and the quality and quantity of DNA were checked using a NanoDrop ND-1000 spectrophotometer (NanoDrop Technologies, Wilmington, DE, USA). For the high-throughput Illumina sequencing, PCR amplifications of the bacterial 16S *rRNA* and fungal ITS genes were performed using the universal primer pairs of 338F/806R [65] and ITS1-1737F/ITS2-2043R, respectively [66]. Both the forward and reverse primers were tagged with an adapter and linker sequence, and 8-bp barcode oligonucleotides were added to distinguish the amplicons from different soil samples. Reaction mixtures (20 μL) contained 4 μL of 5× FastPfu Buffer, 0.25 μL of each primer (10 μM), 2 μL of 2.5 mM dNTPs, 10 ng template DNA, and 0.4 μL FastPfu Polymerase. The PCR protocol was as follows: an initial pre-denaturation at 95 °C for 5 min; 28 cycles of 30 s at 94 °C, 30 s at 55 °C, and 45 s at 72 °C; and a final extension at 72 °C for 10 min. All amplicons were cleaned and pooled in equimolar concentrations in a single tube, after which they were subjected to library preparation, cluster generation, and 250-bp paired-end sequencing on an Illumina MiSeq platform (Illumina Inc., San Diego, CA, USA).

The raw sequences were quality screened and trimmed using the Quantitative Insights into Microbial Ecology (QIIME package version 1.9.1) pipeline [67]. Sequences that fully matched the barcodes were selected and distributed into separate files for the bacterial 16S *rRNA* and fungal ITS genes. Additional sequence processing was performed including quality trimming, demultiplexing, and taxonomic assignments. QIIME quality trimming was performed in accordance with the following criteria: (1) truncated before three consecutive low-quality bases and re-evaluated for length, (2) no ambiguous bases, and (3) the minimum sequence length of 469 bp (16S *rRNA*) and 307 bp (ITS) after trimming. The assembled reads were processed de novo chimera detection conducted with UCHIME [68]. The remaining sequences were additionally screened for frame shifts via HMM-FRAME [69]. Thereafter, the sequence reads from each sample were clustered to provide similarity-based operational taxonomic units (OTUs) that had 97% identity cutoffs [70]. Finally, the sequences of 16S *rRNA* and ITS genes were subjected to a similarity search using the Basic Local Alignment Search Tool (BLAST) of the Silva Release 119 database and UNITE version 6.0 database, respectively [71, 72]. Alpha diversity and Bray-Curtis distances for a principal coordinate analysis of soil microbial community were calculated after rarefying all samples to the same sequencing depth.

### DNA stable isotope probing microcosms and quantitative PCR

To address question 2, we determined the impacts of soil bacterial and fungal networks coupled with keystone taxa on microbial diversity by DNA-stable isotope probing (SIP) microcosms and qPCR. Subsamples were gathered for each treatment from the portion that was stored at − 80 °C. Microcosms with 20 g fresh soil were established in 50 mL sterilized glass serum vials, which were sealed with membrane to allow for air exchange. Microcosms were separated into duplicate groups that were supplemented with 10 mM ^12^C- or ^13^C-labeled glucose, and then incubated at 28 °C for 15 days. The ^13^C-labeled glucose was universally labeled by ^13^C atoms at all carbons (> 99% atom purity, Cambridge Isotope Laboratories, Andover, MA, USA). The isolation of density-gradient fractions of microcosm DNA samples was performed following Dunford and Neufeld [73]. Briefly, an appropriate volume of gradient buffer was added to 2 μg of total DNA and 4.8 mL of 7.163 M cesium chloride (CsCl) density-gradient solution mix in a 15-mL Falcon tube, and the solution was adjusted to a final density of 1.725 g mL^−1^. The solution was transferred into 5 mL Quick-Seal polyallomer centrifuge tubes that were sealed to ensure tube quality. Then, the tubes were weighed, balanced, and loaded into a TLA-120.2 rotor and centrifuged at 45,000 rpm for 44 h under vacuum using a Beckman optima TLX (Beckman Coulter, Inc., Palo Alto, CA, USA). After ultracentrifugation, the solution was immediately separated into 15 fractions from the bottom of each tube using a calibrated infusion pump (New Era Pump System, Inc., Farmingdale, NY, USA). Samples from unlabeled control experiments were always analyzed in parallel as negative controls. ^12^C controls were extremely useful to identify the “heavy” DNA from SIP incubations [74]. The DNA present in each fraction was purified on a MicroCon YM-30 column (Millipore) to remove CsCl and dissolved in 30 μL nuclease-free H_2_O. The pooled heavy density fractions with buoyant densities from 1.725 to 1.750 g mL^−1^ were used for qPCR and Illumina sequencing after incubation of the ^12^C- and ^13^C-labeled glucose. On average, the heavy density fractions in the ^13^C-incubated samples contained over 12 times more DNA than those in the ^12^C-controls for the non-amended microcosms and over 6 times than those for the biochar-amended microcosms.

The copy numbers of 16S *rRNA* and ITS genes in the fractioned DNA of each fraction were quantified by qPCR on a CFX96 Optical Real-Time Detection System (Bio-Rad Laboratories, Hercules, CA, USA) using the same primers as described above. Reaction mixtures (20 μL) contained 1 μL DNA template (1–10 ng), 10 μL 2 × SYBR Premix Ex Taq (TaKaRa, Dalian, China), and 0.5 μM each primer. No-template controls were included with each qPCR run. Standard curves were constructed by a serial dilution (10^2^ to 10^8^ copies) of plasmids harboring 16S *rRNA* and ITS genes. All qPCR assays were run with 3 min initial denaturation at 95 °C, followed by 40 cycles, with plate-reading, of 30 s at 95 °C and 45 s at 60 °C, and then with a final melt-curve step from 72 to 95 °C. The qPCR was performed in triplicate, and amplification efficiencies > 97% were obtained with the *r*^2^ values > 0.99.

### Microbial carbon metabolic profiles and soil metabolic quotient

To address question 3, we measured microbial carbon metabolic activities using Biolog EcoPlates and determined *q*CO_2_ as soil respiration divided by microbial biomass. The capability of soil microbial community to utilize a variety of carbon sources was measured with Biolog EcoPlates (Biolog Inc., Hayward, CA, USA) [75]. The Biolog EcoPlates consisted of 96-well microplates, containing 31 different carbon sources plus a blank well including three replications. Carbon sources were subdivided into six group substrates including carbohydrates, carboxylic acids, amino acids, polymers, phenolic acid, and amines/amides [76]. Soil microorganisms were extracted as follows: 5 g soil (dry weight equivalent) was added to 45 mL sterile 0.85% (*w*/*v*) saline solution. The mixture was shaken for 30 min at 90 rpm and then left to stand for 30 min. Next, 1 mL supernatant was diluted to 20 mL with sterile saline solution. Then, each well of the Biolog EcoPlates was inoculated by 200 μL of the prepared suspension and incubated at 25 °C in the dark for 7 days. The rate of utilization of the carbon sources was pointed by the reduction of tetrazolium violet redox dye, and color development reflecting carbon utilization in the wells was detected by absorbance measurements at 590 nm every 24 h. For the posterior analysis, absorbance at a single time point (96 h) was used, when the asymptote was reached. Optical density (OD_590_) value from each well was corrected by subtracting the control (blank well) values.

Soil respiration was determined according to the modified method described by Chen et al. [77]. Briefly, 25 g soil (dry weight equivalent) was moistened to 60% water-holding capacity and incubated in a 125-mL jar under aerobic conditions at 25 °C. All the jars were kept at the same conditions as the pre-incubation during a period of 7 days. Three replicates of gas samples from each treatment were collected from the headspace of the jars using a gas-tight syringe at 0.5, 1, 3, 5, 7, 10, 15, 20, 25, and 30 days during the incubation period. After gas sampling, stoppers were removed from the jars, and aluminum foil was used to seal the jars again. The CO_2_ concentration was analyzed with a HP 6890 Series gas chromatography (Hewlett Packard, Wilmington, DE, USA) equipped with a flame ionization detector. Three extra jars containing the same weight of pure silica sand were analyzed in parallel as blanks. CO_2_ gas standards were purchased from the National Research Center for Certified Reference Materials, China. The *q*CO_2_ was calculated as soil basal respiration divided by total microbial biomass.

### Statistical analysis

One-way analysis of variance (ANOVA) was performed to assess the effects of fertilization treatments on soil properties, the diversity and abundances of soil bacterial and fungal communities, and microbial carbon metabolism and *q*CO_2_ using Bonferroni’s post hoc test in SPSS 20.0 software (SPSS, Chicago, IL, USA). The significance testing in one-way ANOVA was based on the data from which the samples originated both followed a normal distribution and had the same variances [78]. Principal coordinate analysis (PCoA) was used to evaluate the biochar impacts on the Bray-Curtis distances of the bacterial and fungal community compositions [79]. We conducted the “capscale” function of the R package vegan (version 3.1.2) to calculate the Bray-Curtis dissimilarities for principal coordinate analysis and “permutest” permutation-based testing for the calculation of the significance values [80]. The compositional similarity was calculated as 1 minus the Bray-Curtis dissimilarity.

The non-amended (CK and CF) and biochar-amended (LB, MB, and HB) samples were separately examined for biochar effects on soil bacterial and fungal networks. The OTUs presented either in all non-amended or in all biochar-amended samples were kept for the subsequent network constructions, respectively. The co-occurrence patterns of the bacterial and fungal communities were constructed by calculating multiple correlations and similarities with co-occurrence network (CoNet) inference [81]. We used an ensemble approach based on the four measurements, including Pearson and Spearman correlations and Bray-Curtis and Kullback-Leibler dissimilarities between pairwise OTUs. A valid co-occurrence was considered a statistically robust correlation between taxa when the correlation threshold was above 0.7 and the *P* value was below 0.01. The *P* values were merged using Brown’s method for the four measurements [82] and then adjusted using the Benjamini-Hochberg procedure to reduce the chances of obtaining false-positive results [83]. Network visualization was conducted using Gephi software [84]. Nodes indicated individual microbial taxa (OTUs) in the microbiome network [26]. Network edges represented the pairwise correlations between nodes, suggesting a biologically or biochemically meaningful interactions [12]. The modules were the clusters of closely interconnected nodes (i.e., groups of co-existing or co-evolving microbes) [26]. The microbial networks were searched to identify highly associated nodes (clique-like structures) using Molecular Complex Detection (MCODE) introduced for the Cytoscape platform [85]. The algorithm identifies seeded nodes for expansion by computing a score of local density for each node in the graph. Over 90% accuracy of MCODE predictions yielded, when an overlap score was above 0.2 threshold. The calculated topological characteristics of the bacterial and fungal networks included the following: the numbers of positive and negative correlations, average path length, graph density, network diameter, average clustering coefficient, average connectivity, and modularity. The roles of individual nodes were estimated by two topological parameters: the within-module connectivity *Z,* which quantified to what extent a node connected to other nodes in its own module, and the among-module connectivity *P*, which quantified how well the node connected to different modules [86]. The nodes with either a high value of *Z* or *P* were defined as keystone taxa, including module hubs (*Z* > 0.25, *P* ≤ 0.62; critical to its own module coherence), connectors (*Z* ≤ 0.25, *P* > 0.62; connect modules together and important to network coherence), and network hubs (*Z* > 0.25, *P* > 0.62; vital to both the network and its own module coherence) [87]. For network modules, the module eigengene could summarize the closely connected members within a module [88]. The singular value decomposition of the module expression matrix was used to represent the module eigengene networks [89]. The module eigengene of a module was defined as the first principal component of the standardized module expression data [90]. Then, the relationships between soil properties, microbial diversity, network module eigengenes, and SOC mineralization (microbial carbon metabolism and *q*CO_2_) were evaluated using Spearman’s rank correlation test.

Random forest modeling was used to quantitatively assess the important predictors of carbohydrate catabolism and *q*CO_2_ involving soil properties and the microbial community. Soil properties included soil pH, SMC, SOC, total nitrogen, total phosphorus, total potassium, available nitrogen, available phosphorus, available potassium, and cation exchange capacity, while the microbial community included the biomass, diversity, composition, and network of soil bacterial and fungal communities. The bacterial and fungal biomass were characterized by bacterial and fungal PLFAs. The bacterial and fungal diversities were represented by the Shannon index based on the rarified same sequencing depth. The compositions of soil bacterial and fungal communities were indicated by the first principal coordinates (PCoA1). The bacterial and fungal networks were represented by the module eigengenes that were significantly related to diversity and carbohydrate metabolism. The importance of each factor was evaluated by the increase in the mean square error between the observed and predicted values when the predictor was randomly permuted [91]. This accuracy of importance was measured for each tree and was averaged across the forest. Accuracy of importance was estimated for each observation using the left-out individual predictions (called “out-of-bag” estimation) and then averaged over all observations [92]. These analyses were conducted using the randomForest package [93], and the significance of the model and predictor importance was determined using the A3 and rfPermute packages, respectively [94, 95]. Structural equation modeling (SEM) was applied to determine the direct and indirect contributions of soil properties and microbial community to carbohydrate catabolism and *q*CO_2_ [96]. SEM analysis was conducted via the robust maximum likelihood evaluation method using AMOS 20.0 (AMOS IBM, USA). The SEM fitness was examined on the basis of a non-significant chi-square test (*P* > 0.05), the goodness-of-fit index (GFI), and the root mean square error of approximation (RMSEA) [97].

## Additional file

Additional file 1: **Figure S1.** Soil water characteristic curves (a) and equation diameter of pore versus water content (b, d-θ) curves under nonamended and biochar-amended treatments in the field experimnet. **Figure S2.** Effects of biochar amendments on total phospholipid fatty acid (PLFA) and various microbial specific groups in the field experimnet. **Figure S3.** Taxonomic compositions of bacterial (a) and fungal (b) communities under non-amended and biochar-amended treatments in the field experimnet. **Figure S4.** Biochar amendments alter the bacterial (a) and fungal (b) community composition in the field experimnet. **Figure S5.** Mean predictor importance of soil properties and the biomass, diversity, composition, and networks of the bacterial and fungal communities on carbohydrate utilization (a) and soil metabolic quotient (b) based on random forest modeling. **Figure S6.** Biochar treatments alter the bacterial (a, b) and fungal (c, d) diversity in the conducted stable isotope probing microcosms. **Figure S7.** Taxonomic compositions of bacterial (a) and fungal (b) communities in the conducted stable isotope probing microcosms. **Table S1.** Soil physicochemical properties condition under five treatments. **Table S2.** Topological properties of co-occurring bacterial and fungal networks obtained under biochar non-amended and amended treatments in the field experiment and stable isotope probing incubations. **Table S3.** Correlations of soil properties, the biomass and diversity of bacterial and fungal communities, carbohydrate catabolism, and soil metabolic quotient (*q*CO_2_). (DOCX 1022 kb)
