# Correction to: Competitive interaction with keystone taxa induced negative priming under biochar amendments

**DOI:** 10.1186/s40168-019-0765-8

**Published:** 2019-11-22

**Authors:** Lijun Chen, Yuji Jiang, Chao Liang, Yu Luo, Qinsong Xu, Cheng Han, Qiguo Zhao, Bo Sun

**Affiliations:** 1 State Key Laboratory of Soil and Sustainable Agriculture, Institute of SoilScience, Chinese Academy of Sciences, No. 71 East Beijing Road, Nanjing, 210008 China; 2 Institute of Applied Ecology, Chinese Academy of Sciences, Shenyang, 110016 China; 3 Zhejiang Provincial Key Laboratory of AgriculturalResources and Environment, Institute of Soil and Water Resources andEnvironmental Science, Zhejiang University, Hangzhou, 310058 China; 4 College of Life Science, Nanjing Normal University, Nanjing, 210023 China; 5 School of Geography Science, Nanjing Normal University, Nanjing, 210023 China; 6 University of Chinese Academy of Sciences, Beijing, 100049 China

**Correction to: Microbiome (2019) 7:77**

**https://doi.org/10.1186/s40168-019-0693-7**

Following publication of the original article [1], the authors reported an error in the Additional file 1. The deleted Additional file 2 (**SUPPLEMENTARY FIGURES FOR REVIEW**) was mistakenly uploaded as the Additional file 1. The correct file, **SUPPLEMENTARY FIGURES FOR PUBLICATION,** is available here.

The publishers apologise for this error.

## Supplementary information

**Additional file 1: Figure S1.** Soil water characteristic curves (a) and equation diameter of pore versus water content (b, d-θ) curves under nonamended and biochar-amended treatments in the field experimnet. **Figure S2.** Effects of biochar amendments on total phospholipid fatty acid (PLFA) and various microbial specific groups in the field experimnet. **Figure S3.** Taxonomic compositions of bacterial (a) and fungal (b) communities under non-amended and biochar-amended treatments in the field experimnet. **Figure S4.** Biochar amendments alter the bacterial (a) and fungal (b) community composition in the field experimnet. **Figure S5.** Mean predictor importance of soil properties and the biomass, diversity, composition, and networks of the bacterial and fungal communities on carbohydrate utilization (a) and soil metabolic quotient (b) based on random forest modeling. **Figure S6.** Biochar treatments alter the bacterial (a, b) and fungal (c, d) diversity in the conducted stable isotope probing microcosms. **Figure S7.** Taxonomic compositions of bacterial (a) and fungal (b) communities in the conducted stable isotope probing microcosms. **Table S1.** Soil physicochemical properties condition under five treatments. **Table S2.** Topological properties of co-occurring bacterial and fungal networks obtained under biochar non-amended and amended treatments in the field experiment and stable isotope probing incubations. **Table S3.** Correlations of soil properties, the biomass and diversity of bacterial and fungal communities, carbohydrate catabolism, and soil metabolic quotient (*q*CO_2_).
