# Supplementary material for: Competitive interaction with keystone taxa induced negative priming under biochar amendments
Source: Microbiome. 2019 May 20;7:77. doi: 10.1186/s40168-019-0693-7 (PMC6526607; doi:10.1186/s40168-019-0693-7)
Supplement: Supplementary file 1 — Figure S1. Soil water characteristic curves (a) and equation diameter of pore versus water content (b, d-θ) curves under nonamended and biochar-amended treatments in the field experimnet. Figure S2. Effects of biochar amendments on total phospholipid fatty acid (PLFA) and various microbial specific groups in the field experimnet. Figure S3. Taxonomic compositions of bacterial (a) and fungal (b) communities under non-amended and biochar-amended treatments in the field experimnet. Figure S4. Biochar amendments alter the bacterial (a) and fungal (b) community composition in the field experimnet. Figure S5. Mean predictor importance of soil properties and the biomass, diversity, composition, and networks of the bacterial and fungal communities on carbohydrate utilization (a) and soil metabolic quotient (b) based on random forest modeling. Figure S6. Biochar treatments alter the bacterial (a, b) and fungal (c, d) diversity in the conducted stable isotope probing microcosms. Figure S7. Taxonomic compositions of bacterial (a) and fungal (b) communities in the conducted stable isotope probing microcosms. Table S1. Soil physicochemical properties condition under five treatments. Table S2. Topological properties of co-occurring bacterial and fungal networks obtained under biochar non-amended and amended treatments in the field experiment and stable isotope probing incubations. Table S3. Correlations of soil properties, the biomass and diversity of bacterial and fungal communities, carbohydrate catabolism, and soil metabolic quotient (qCO2). (DOCX 1022 kb) [file 40168_2019_693_MOESM1_ESM.docx]

**SUPPLEMENTARY FIGURES FOR REVIEW**


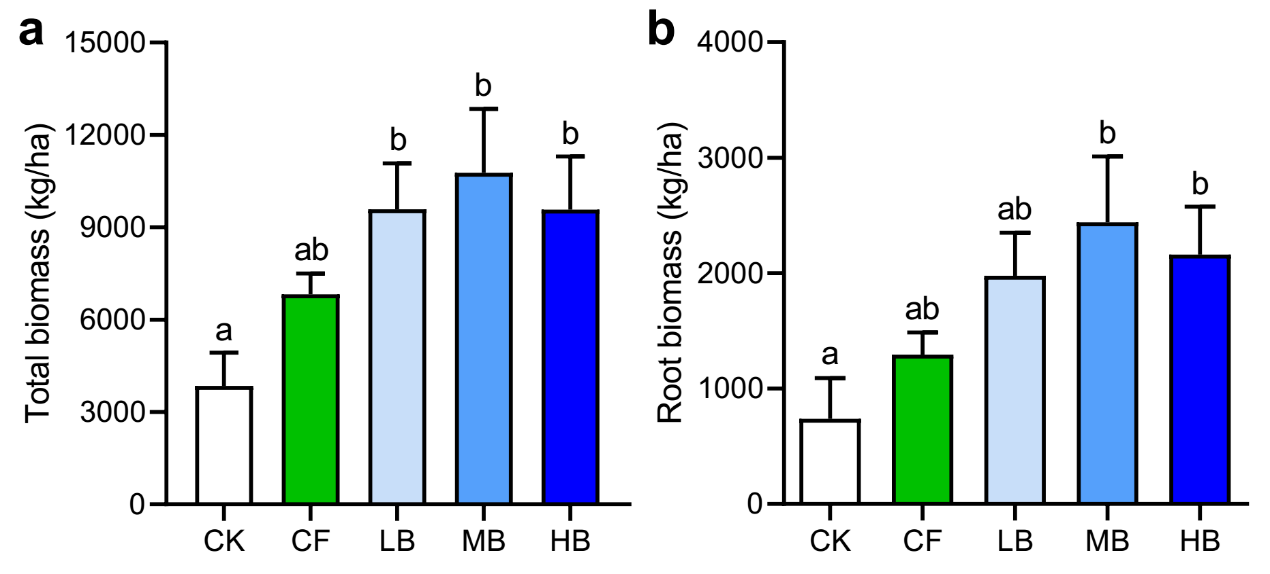


**Figure R1** Effects of the biochar amendments on total plant biomass and root biomass in the field experiment. Bars with different lowercase letters indicate statistical significant differences (*P* < 0.05) as revealed by Bonferroni’s post-hoc tests. CK, no fertilizer; CF, conventional fertilization; LB, low biochar with 2400 kg ha^–1^ y^−1^; MB, medium biochar with 7200 kg ha^−1^ y^−1^; HB, high biochar with 12000 kg ha^−1^ y^−1^.


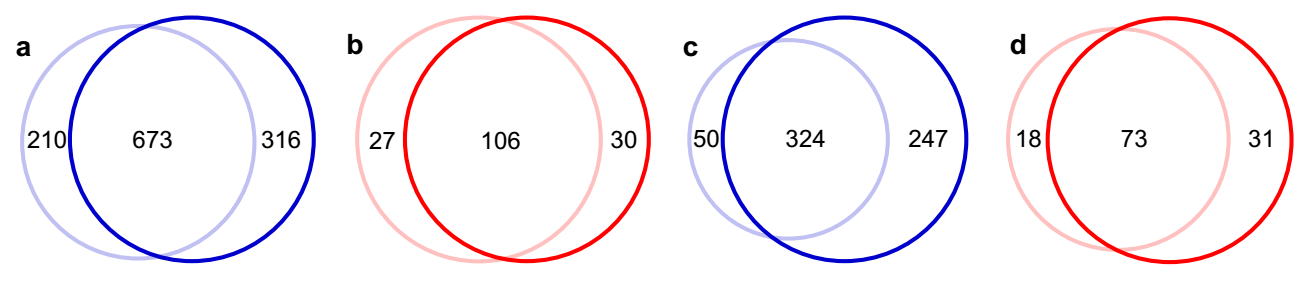
**Figure R2** Venn diagram shows the specific and core OTUs of the bacterial (blue) and fungal (red) communities in the field experiments (a, b) and the microcosm incubations (c, d). The light and dark color circles indicate the nonamended (CF and CK) and biochar-amended networks (LB, MB, and HB), respectively.
