# Supplementary material for: Correction to: Competitive interaction with keystone taxa induced negative priming under biochar amendments
Source: Microbiome. 2019 Nov 22;7:150. doi: 10.1186/s40168-019-0765-8 (PMC6874812; doi:10.1186/s40168-019-0765-8)
Supplement: Supplementary file 1 — Additional file 1: Figure S1. Soil water characteristic curves (a) and equation diameter of pore versus water content (b, d-θ) curves under nonamended and biochar-amended treatments in the field experimnet. Figure S2. Effects of biochar amendments on total phospholipid fatty acid (PLFA) and various microbial specific groups in the field experimnet. Figure S3. Taxonomic compositions of bacterial (a) and fungal (b) communities under non-amended and biochar-amended treatments in the field experimnet. Figure S4. Biochar amendments alter the bacterial (a) and fungal (b) community composition in the field experimnet. Figure S5. Mean predictor importance of soil properties and the biomass, diversity, composition, and networks of the bacterial and fungal communities on carbohydrate utilization (a) and soil metabolic quotient (b) based on random forest modeling. Figure S6. Biochar treatments alter the bacterial (a, b) and fungal (c, d) diversity in the conducted stable isotope probing microcosms. Figure S7. Taxonomic compositions of bacterial (a) and fungal (b) communities in the conducted stable isotope probing microcosms. Table S1. Soil physicochemical properties condition under five treatments. Table S2. Topological properties of co-occurring bacterial and fungal networks obtained under biochar non-amended and amended treatments in the field experiment and stable isotope probing incubations. Table S3. Correlations of soil properties, the biomass and diversity of bacterial and fungal communities, carbohydrate catabolism, and soil metabolic quotient (qCO2). [file 40168_2019_765_MOESM1_ESM.docx]

**SUPPLEMENTARY INFORMATION FOR**

**Competitive network structure with keystone taxa induced negative priming under biochar amendments**

Lijun Chen^a,f,1^, Yuji Jiang^a,1,^*, Chao Liang^b^, Yu Luo^c^, Qinsong Xu^d^, Cheng Han^e^, Qiguo Zhao^a^, Bo Sun^a,^ *

**Corresponding authors:**

Bo Sun E-mail: bsun@issas.ac.cn.

Institute of Soil Science, Chinese Academy of Science, No. 71 East Beijing Road, Nanjing, 210008, China. Tel.: +86 25 86881282; Fax: +86 25 86881000.

Yuji Jiang E-mail: yjjiang@issas.ac.cn.

Institute of Soil Science, Chinese Academy of Science, No. 71 East Beijing Road, Nanjing, 210008, China. Tel.: +86 25 86881245; Fax: +86 25 86881000.

**Supplementary Figures S1–S7**

**Figure S1** Soil water characteristic curves and equation diameter of pore versus water content (b, d-θ) curves under nonamended and biochar-amended treatments in the field experimnet.

**Figure S2** Effects of biochar amendments on total phospholipid fatty acid (PLFA) and various microbial specific groups in the field experimnet.

**Figure S3** Taxonomic compositions of bacterial and fungal communities under nonamended and biochar-amended treatments in the field experimnet.

**Figure S4** Biochar amendments alter the bacterial and fungal community composition in the field experimnet.

**Figure S5** Mean predictor importance of soil properties, and the biomass, diverisity, composition and networks of the bacterial and fungal communities on carbohydrate utilizaiton and soil metabolic quotient based on random forest modelling.

**Figure S6** Biochar treatments alter the bacterial and fungal diversity in the conducted stable isotope probing microcosms.

**Figure S7** Taxonomic compositions of bacterial (a) and fungal (b) communities in the conducted stable isotope probing microcosms.

**Supplementary Tables S1–S3**

**Table S1** Soil physicochemical properties condition under five treatments.

**Table S2** Topological properties of co-occurring bacterial and fungal networks obtained under biochar nonamended and amended treatments in the field experiment and stable isotope probing incubations.

**Table S3** Correlations of soil properties, the biomass and diversity of bacterial and fungal communities, carbohydrate catabolism, and soil metabolic quotient (*q*CO_2_).

**Supplementary Figures**

**
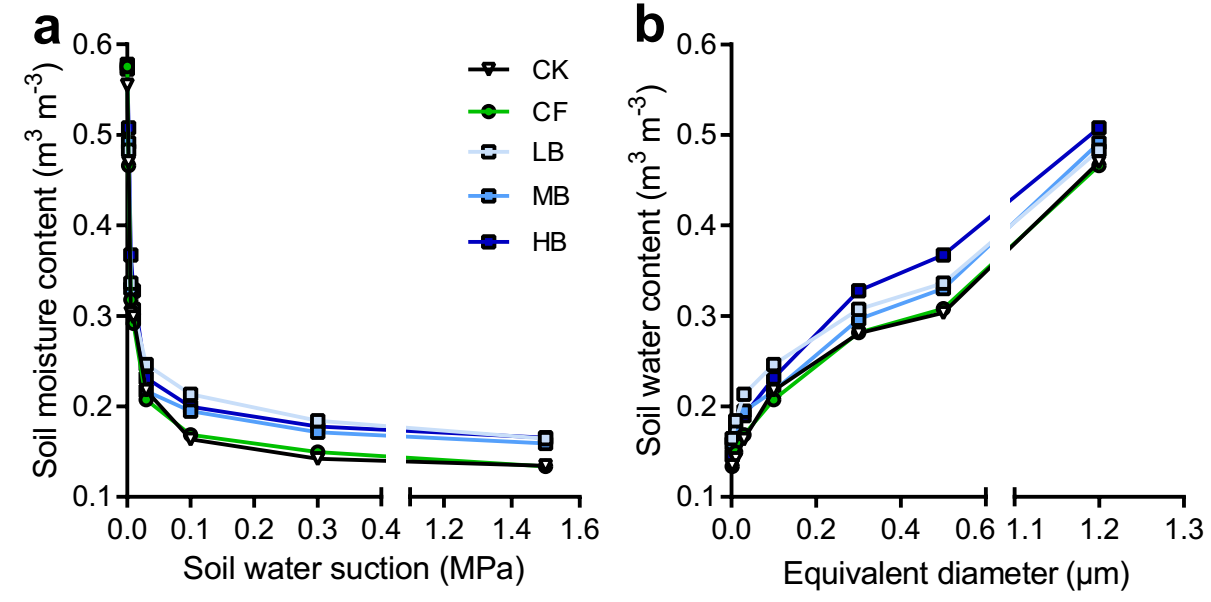
**

**Figure S1** Soil water characteristic curves (a) and equation diameter of pore versus water content (b, d-θ) curves under nonamended and biochar-amended treatments in the field experimnet. CK, no fertilizer; CF, conventional fertilization; LB, low biochar with 2400 kg ha^–1^ y^−1^; MB, medium biochar with 7200 kg ha^−1^ y^−1^; HB, high biochar with 12000 kg ha^−1^ y^−1^.


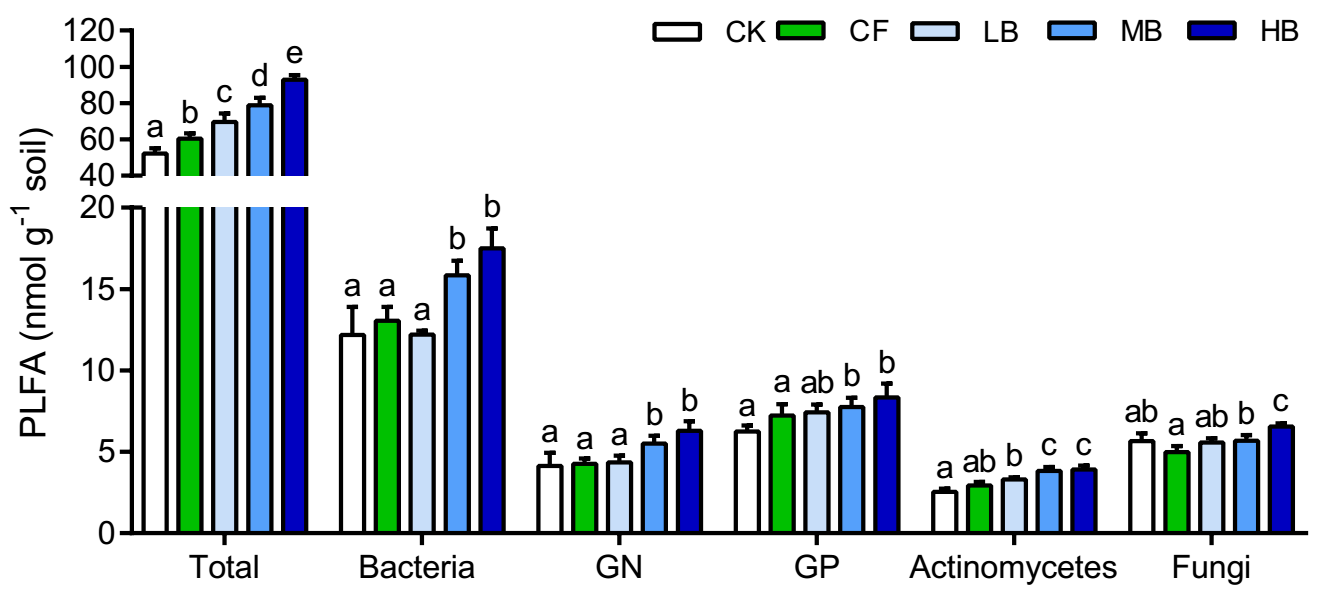


**Figure S2** Effects of biochar amendments on total phospholipid fatty acid (PLFA) and various microbial specific groups in the field experimnet, including bacteria, Gram-negative bacteria (GN), Gram-positive bacteria (GP), actinomycetes, and fungi. Bars with different lowercase letters indicate statistical significant differences (*P* < 0.05) as revealed by Bonferroni’s post-hoc tests. CK, no fertilizer; CF, conventional fertilization; LB, low biochar with 2400 kg ha^–1^ y^−1^; MB, medium biochar with 7200 kg ha^−1^ y^−1^; HB, high biochar with 12000 kg ha^−1^ y^−1^.


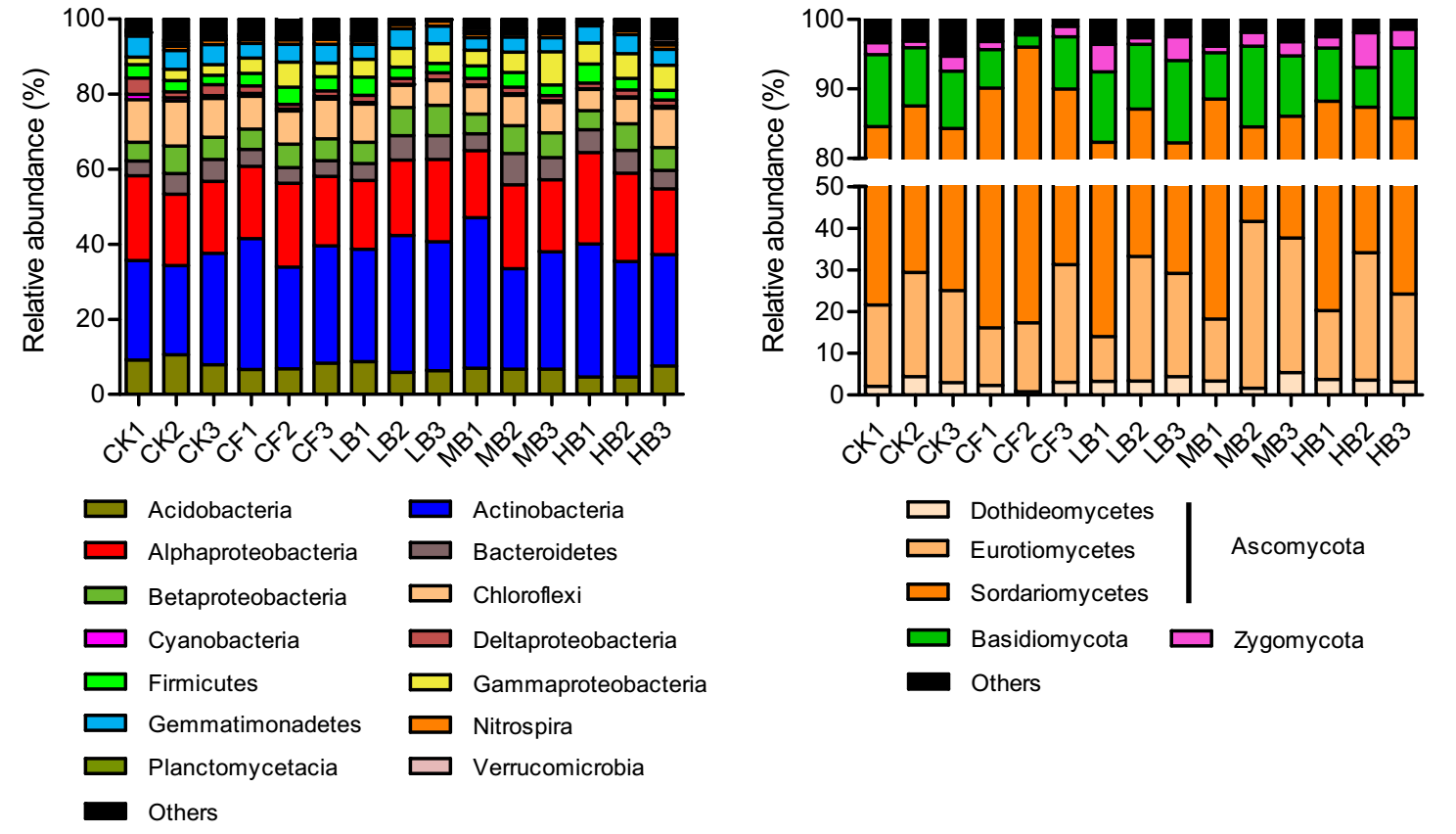


**Figure S3** Taxonomic compositions of bacterial (a) and fungal (b) communities under nonamended and biochar-amended treatments in the field experimnet. The abundances of bacterial and fungal communities are based on the proportional frequencies of 16S *rRNA* and ITS sequences, respectively. CK, no fertilizer; CF, conventional fertilization; LB, low biochar with 2400 kg ha^–1^ y^−1^; MB, medium biochar with 7200 kg ha^−1^ y^−1^; HB, high biochar with 12000 kg ha^−1^ y^−1^.


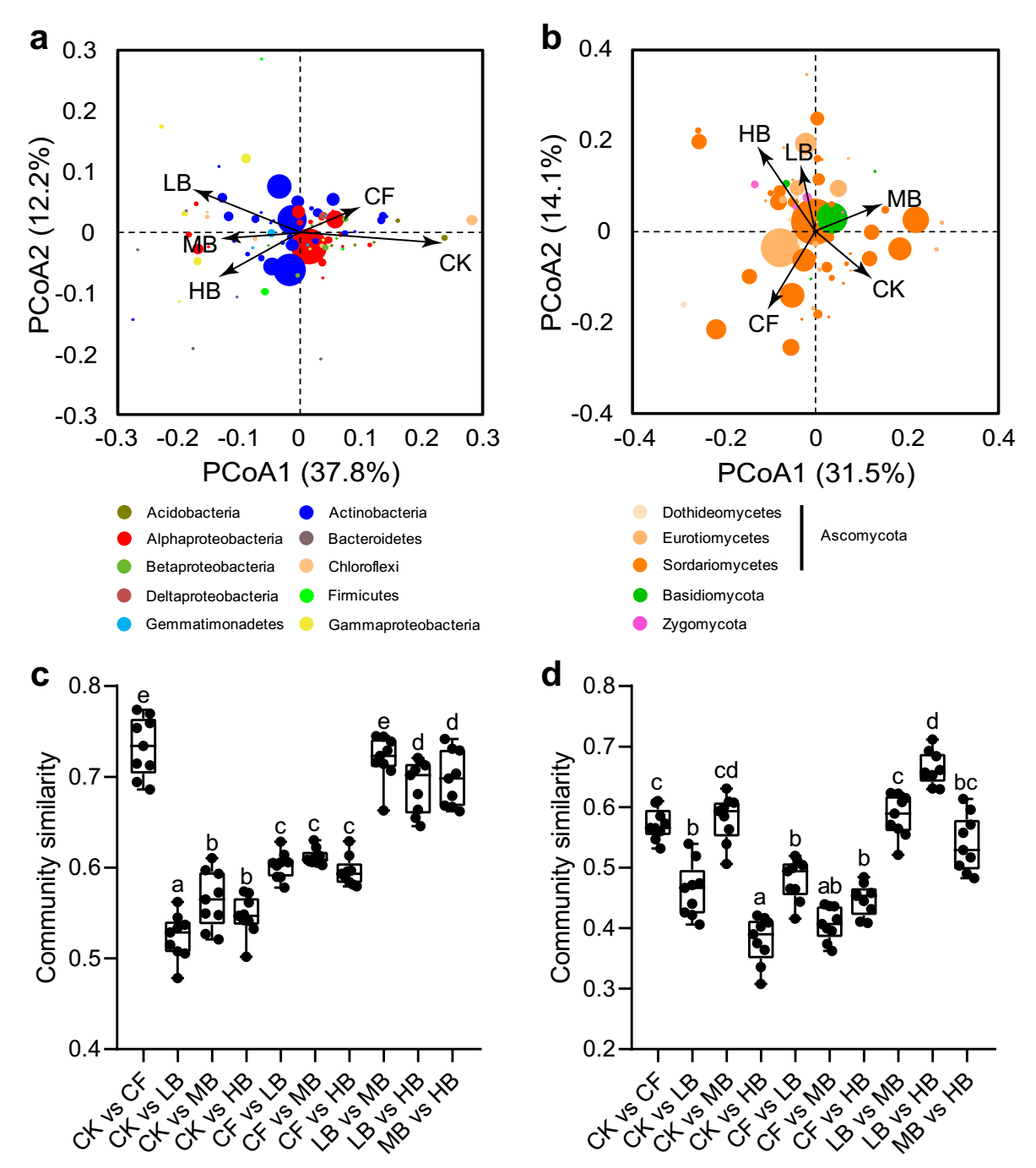


**Figure S4** Biochar amendments alter the bacterial and fungal community composition in the field experimnet. The dominant OTUs (relative abundance > 0.1%) scores in the bacterial (a) and fungal (b) community by principal coordinate analysis (PCoA), which are constrained by biochar amendments and based on Bray-Curtis distances among all the samples. The arrows point to the centroid of the constrained factor. Circle sizes correspond to the abundance of bacterial and fungal OTUs, and colors are assigned to different bacterial and fungal phyla/classes. The compositional similarity of bacterial (c) and fungal (d) communities between biochar amendments and their compartments (CK and CF). The similarity is calculated as 1 minus the Bray–Curtis dissimilarity. CK, no fertilizer; CF, conventional fertilization; LB, low biochar with 2400 kg ha^–1^ y^−1^; MB, medium biochar with 7200 kg ha^−1^ y^−1^; HB, high biochar with 12000 kg ha^−1^ y^−1^.


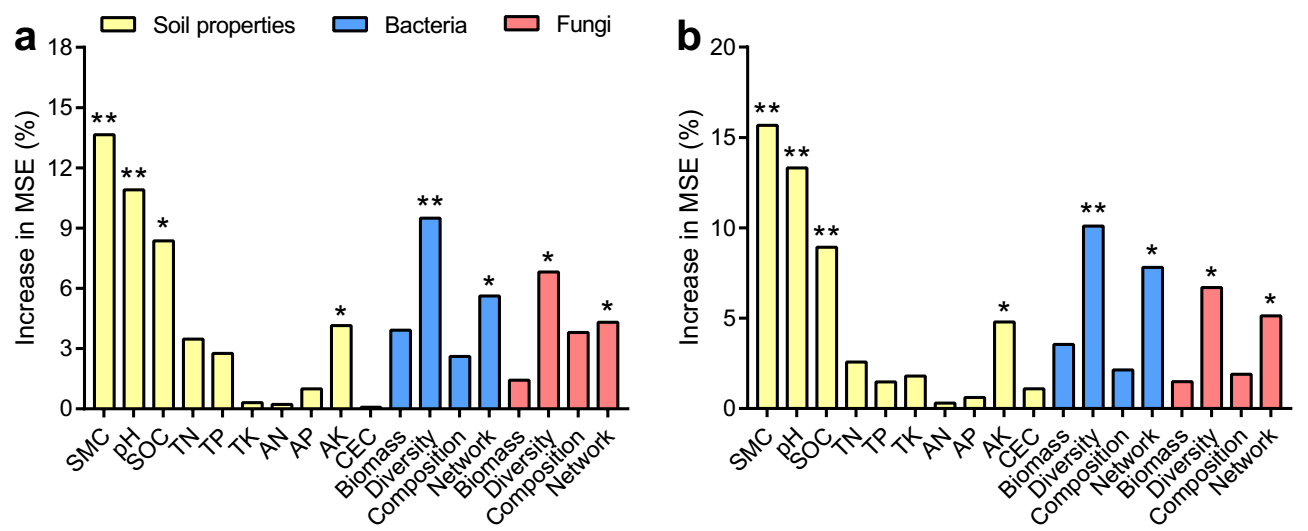


**Figure S5** Mean predictor importance (% of increased mean square error, MSE) of soil properties, and the biomass, diverisity, composition and networks of the bacterial and fungal communities on carbohydrate utilization (a) and soil metabolic quotient (b) based on random forest modelling. The bacterial and fungal biomass are characterized by bacterial and fungal PLFAs. The bacterial and fungal diversity are used to represented by Shannon index based on the rarified same sequencing depth. The composition of soil bacterial and fungal communities are represented by the first principal coordinates (PCoA1 explained 37.8% and 31.5% of the variations, see Figure S4). The bacterial and fungal networks are represented by the module eigengenes that are significantly related to diversity and carbohydrate metabolism. The models for carbohydrate utilizaiton and soil metabolic quotient were significant at the 0.01 level with R^2^ = 0.75 and 0.73, respectively. SMC, soil moisture capacity; SOC, soil organic carbon; TN, total nitrogen; TP, total phosphorus; TK, total potassium; AN, available nitrogen; AP, available phosphorus; AK, available potassium; CEC, cation exchange capacity. Significance level of predictors is as follows: * *P* < 0.05; ** *P* < 0.01.


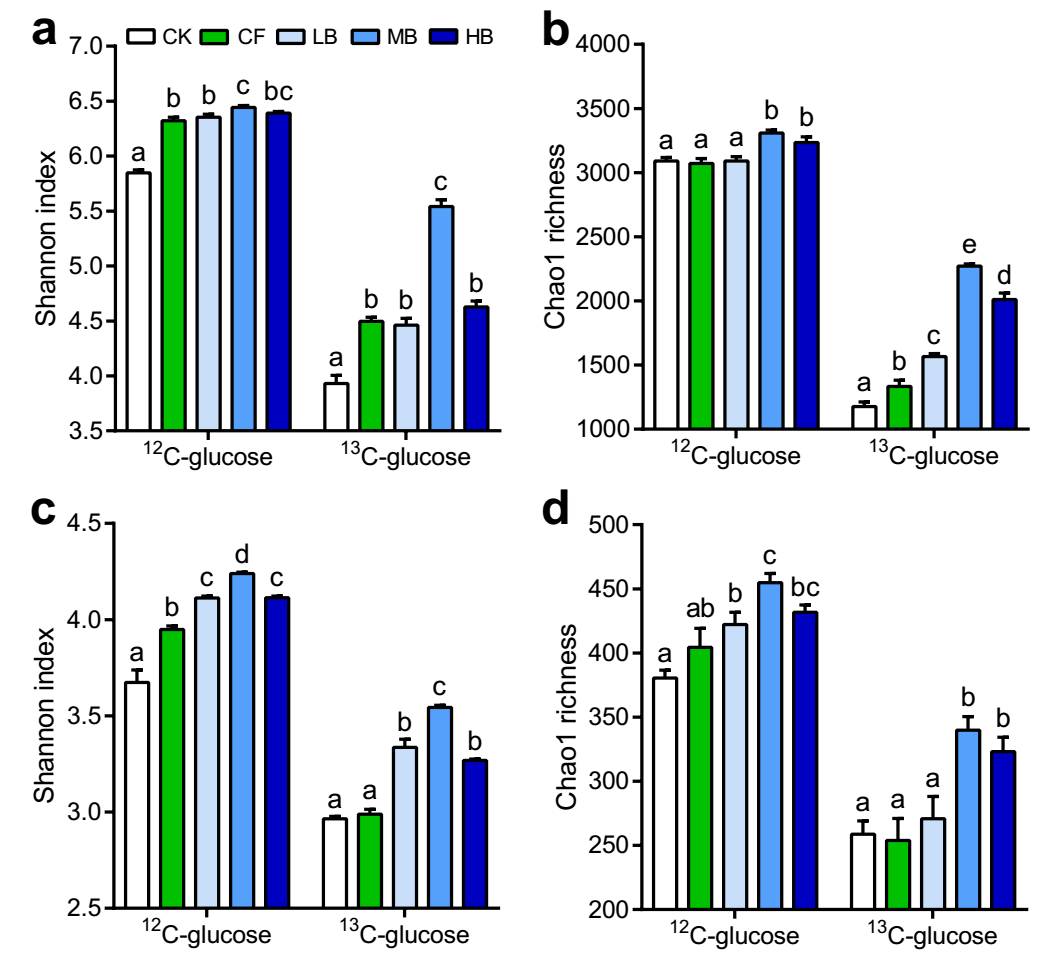


**Figure S6** Biochar treatments alter the bacterial (a, b) and fungal (c, d) diversity in the conducted stable isotope probing microcosms. Calculation of Shannon index and Chao1 richness is based on OTU tables rarified to the same sequencing depth. Bars with different lowercase letters are significantly different (*P* < 0.05) by Bonferroni's post-hoc tests. CK, no fertilizer; CF, conventional fertilization; LB, low biochar with 2400 kg ha^–1^ y^−1^; MB, medium biochar with 7200 kg ha^−1^ y^−1^; HB, high biochar with 12000 kg ha^−1^ y^−1^.


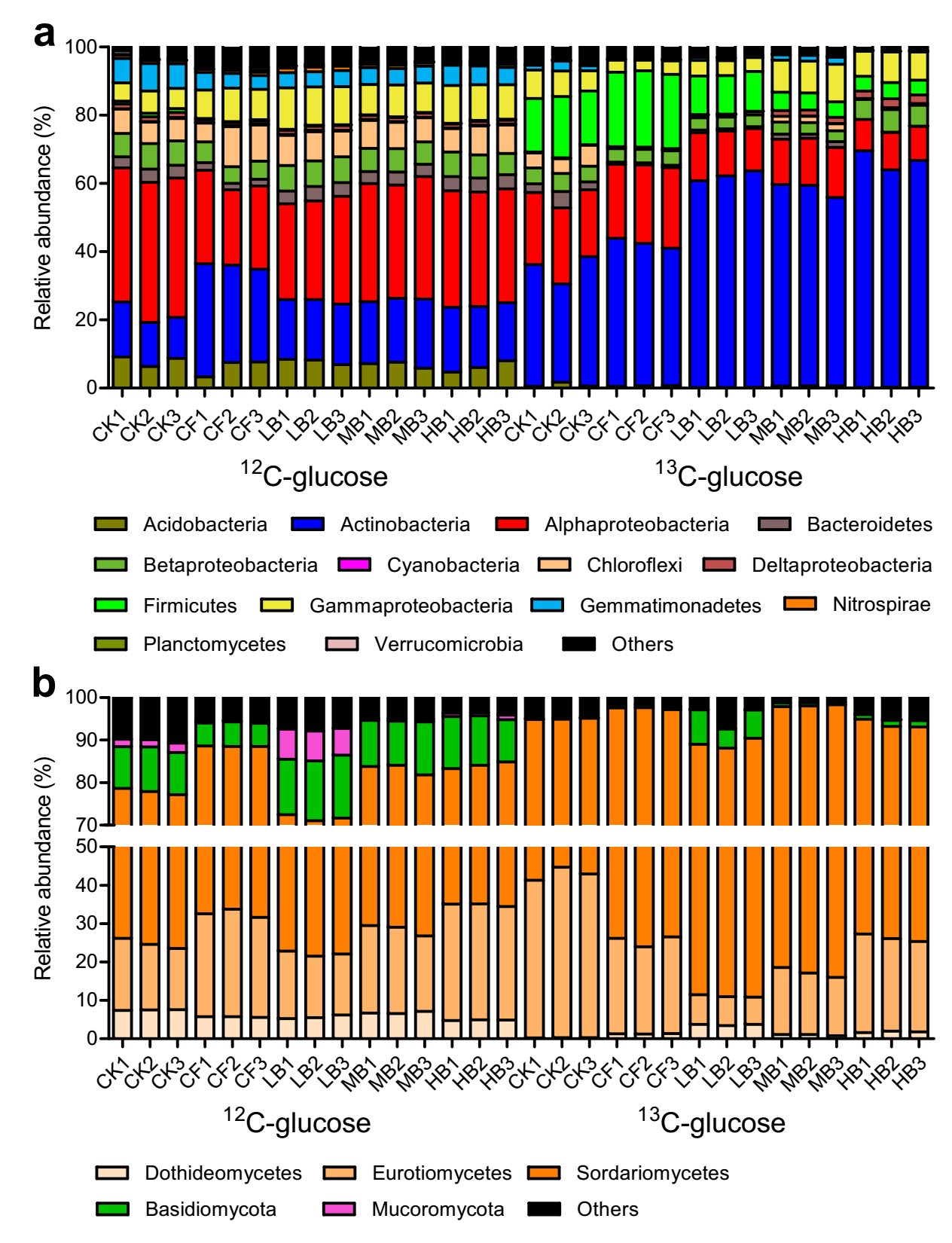


**Figure S7** Taxonomic compositions of bacterial (a) and fungal (b) communities in the conducted stable isotope probing microcosms. The abundances of bacterial and fungal communities are based on the proportional frequencies of 16S *rRNA* and ITS sequences. CK, no fertilizer; CF, conventional fertilization; LB, low biochar with 2400 kg ha^–1^ y^−1^; MB, medium biochar with 7200 kg ha^−1^ y^−1^; HB, high biochar with 12000 kg ha^−1^ y^−1^.

**Supplementary Tables**

**Table S1** Soil physicochemical properties condition under five treatments^a^.

|  | CK | CF | LB | MB | HB |
| --- | --- | --- | --- | --- | --- |
| pH | 6.02±0.05b | 5.71±0.04a | 6.14±0.04b | 6.39±0.01c | 6.57±0.02c |
| SOC (g kg^−1^) | 5.77±0.26a | 6.06±0.13a | 7.97±0.05b | 14.99±0.63c | 16.29±0.30c |
| TN (g kg^−1^) | 0.68±0.01a | 0.80±0.01b | 0.81±0.02b | 1.08±0.03c | 1.12±0.01c |
| TP (g kg^−1^) | 0.42±0.02a | 0.45±0.03ab | 0.48±0.02bc | 0.51±0.01c | 0.52±0.01c |
| TK (g kg^−1^) | 20.74±0.58a | 21.29±0.59ab | 22.48±0.40bc | 22.45±0.37bc | 23.55±0.15c |
| AN (mg kg^−1^) | 50.23±1.29ab | 52.68±2.16b | 49.89±0.97ab | 47.78±0.89a | 47.77±0.56a |
| AP (mg kg^−1^) | 36.13±2.48a | 47.03±0.86b | 52.35±1.35bc | 60.61±1.23c | 60.75±2.05c |
| AK (mg kg^−1^) | 183.33±7.94a | 283.33±7.73b | 316.67±8.45bc | 541.67±11.37c | 550±11.58c |
| CEC (cmol kg^−1^) | 13.80±1.20a | 13.50±0.75a | 14.01±1.63a | 13.70±0.32a | 14.79±1.07a |
| Bulk density (g cm^−3^) | 1.38±0.01c | 1.29±0.01c | 1.21±0.01b | 1.19±0.01b | 1.14±0.01a |

**a.** Values are the means (n=3) ± the standard error of the mean. Values in the same column followed by a lowercase letter indicate significant differences (*P* < 0.05) revealed by Bonferroni’s post hoc test. CK, no fertilizer; CF, conventional fertilization; LB, low biochar with 2400 kg ha^–1^ y^−1^; MB, medium biochar with 7200 kg ha^−1^ y^−1^; HB, high biochar with 12000 kg ha^−1^ y^−1^. SOC, soil organic carbon; TN, total nitrogen; TP, total phosphorus; TK, total potassium; AN, available nitrogen; AP, available phosphorus; AK, available potassium; CEC, cation exchange capacity.

**Table S2** Topological properties of co-occurring bacterial and fungal networks obtained under biochar nonamended and amended treatments in the field experiment and stable isotope probing microcosms^a^.

| Network metrics | Bacterial community | |  | Fungal community | |
| --- | --- | --- | --- | --- | --- |
|  | nonamended | amended |  | nonamended | amended |
| **Empirical networks** |  |  |  |  |  |
| Number of nodes | 59 (47) | 77 (65) |  | 50 (38) | 58 (46) |
| Number of edges | 82 (48) | 128 (80) |  | 71 (41) | 88 (57) |
| Number of positive correlations | 62 (38) | 92 (52) |  | 62 (30) | 59 (33) |
| Number of negative correlations | 20 (10) | 36 (28) |  | 9 (11) | 29 (24) |
| Average path length (APL) | 2.824 | 1.837 |  | 3.436 | 4.942 |
| Graph Density | 0.025 | 0.048 |  | 0.062 | 0.057 |
| Network diameter | 7 | 5 |  | 10 | 13 |
| Average clustering coefficient (*avgCC*) | 0.161 | 0.185 |  | 0.535 | 0.566 |
| Average connectivity (*avgK*) | 2.780 | 3.325 |  | 2.840 | 3.034 |
| Number of modules^b^ | 6 | 5 |  | 4 | 6 |
| Modularity (M) | 0.655 | 0.695 |  | 0.757 | 0.749 |

**a.** The numbers in parentheses indicate the nodes and edges observed in the stable isotope probing microcosms.

**b.** The number of modules with ≥5 nodes in networks.

**Table S3** Correlations of soil properties, the biomass and diversity of bacterial and fungal communities, carbohydrate catabolism, and soil metabolic quotient (*q*CO_2_)^a^.

|  | SMC | pH | SOC | AP | Carbohydrate | *q*CO_2_ |
| --- | --- | --- | --- | --- | --- | --- |
| **Bacterial community** | | | | | | |
| Bacterial biomass | 0.788*** | 0.673** | 0.593* | 0.508 | −0.389 | −0.484 |
| Shannon index | 0.824*** | 0.867*** | 0.711** | 0.603* | −0.838*** | −0.898*** |
| Chao1 richness | 0.839*** | 0.920*** | 0.699** | 0.584* | −0.720** | −0.927*** |
| **Fungal community** | | | | | | |
| Fungal biomass | 0.684** | 0.563* | 0.634* | 0.677** | −0.490 | −0.271 |
| Shannon index | 0.732** | 0.937*** | 0.589* | 0.664** | −0.801*** | −0.826*** |
| Chao1 richness | 0.813*** | 0.885*** | 0.793*** | 0.825*** | −0.868*** | −0.844*** |

**a.** Soil properties include pH, soil moisture capacity (SMC), soil organic carbon (SOC), and available phosphorous (AP). *** *P* < 0.001; ** *P* < 0.01; * *P* < 0.05.
